# The round-robin approach applied to nanoinformatics: consensus prediction of nanomaterials zeta potential

**DOI:** 10.3762/bjnano.15.121

**Published:** 2024-11-29

**Authors:** Dimitra-Danai Varsou, Arkaprava Banerjee, Joyita Roy, Kunal Roy, Giannis Savvas, Haralambos Sarimveis, Ewelina Wyrzykowska, Mateusz Balicki, Tomasz Puzyn, Georgia Melagraki, Iseult Lynch, Antreas Afantitis

**Affiliations:** 1 NovaMechanics MIKE, Piraeus 18545, Greece; 2 Entelos Institute, Larnaca 6059, Cyprus; 3 Drug Theoretics and Cheminformatics (DTC) Lab, Department of Pharmaceutical Technology, Jadavpur University, Kolkata 700 032, India; 4 School of Chemical Engineering, National Technical University of Athens, 9 Iroon Polytechniou, 15780, Athens, Greece; 5 QSAR Lab, Trzy Lipy 3, 80-172 Gdańsk, Poland; 6 University of Gdańsk, Faculty of Chemistry, Laboratory of Environmental Chemoinformatics, Wita Stwosza 63, 80-308 Gdańsk, Poland; 7 Division of Physical Sciences and Applications, Hellenic Military Academy, Vari 16672, Greece; 8 School of Geography, Earth and Environmental Sciences, University of Birmingham, Edgbaston, B15 2TT Birmingham, United Kingdom; 9 NovaMechanics Ltd., Nicosia 1070, Cyprus

**Keywords:** consensus modelling, read-across, QSPR, round-robin test, zeta potential

## Abstract

A key step in building regulatory acceptance of alternative or non-animal test methods has long been the use of interlaboratory comparisons or round-robins (RRs), in which a common test material and standard operating procedure is provided to all participants, who measure the specific endpoint and return their data for statistical comparison to demonstrate the reproducibility of the method. While there is currently no standard approach for the comparison of modelling approaches, consensus modelling is emerging as a “modelling equivalent” of a RR. We demonstrate here a novel approach to evaluate the performance of different models for the same endpoint (nanomaterials’ zeta potential) trained using a common dataset, through generation of a consensus model, leading to increased confidence in the model predictions and underlying models. Using a publicly available dataset, four research groups (NovaMechanics Ltd. (NovaM)-Cyprus, National Technical University of Athens (NTUA)-Greece, QSAR Lab Ltd.-Poland, and DTC Lab-India) built five distinct machine learning (ML) models for the in silico prediction of the zeta potential of metal and metal oxide-nanomaterials (NMs) in aqueous media. The individual models were integrated into a consensus modelling scheme, enhancing their predictive accuracy and reducing their biases. The consensus models outperform the individual models, resulting in more reliable predictions. We propose this approach as a valuable method for increasing the validity of nanoinformatics models and driving regulatory acceptance of in silico new approach methodologies for the use within an “Integrated Approach to Testing and Assessment” (IATA) for risk assessment of NMs.

## Introduction

Nanotechnology, defined as the ability to manipulate matter at the nanoscale, has opened an array of possibilities for multiple applications that take advantage of the unique properties of nanomaterials (NMs). From targeted drug delivery to environmental sensing, the versatility of NMs makes them ideal candidates for a broad range of innovative applications [1]. However, the complexity and unique properties of these materials also present significant challenges, especially when it comes to the assessment of their potential adverse effects. The integration of in silico new approach methodologies (NAMs) within the area of nanotechnology has created a plethora of possibilities for the assessment of NM properties and toxicity to support and/or substitute traditional experimental methodologies [2–3].

The field of nanoinformatics covers a broad range of computational and data-driven methodologies for the exposure, hazard, and risk assessment of NMs, such as quantitative structure–activity relationship models adapted to the specificities of NMs (nanoQSAR) and grouping/read-across models, specifically developed to accurately predict NMs’ properties when small datasets are available [4–6]. These in silico methodologies can be used in the early steps of the “safe-and-sustainable by design” framework and in the development of novel NMs to filter out unpromising candidates and prioritize NMs with desired properties. The rational use of in silico methods allows for the identification of potential hazardous effects caused by NMs’ interactions with biological systems with a simultaneous decrease of workload, cost, research duration, and use of laboratory animals. Several computational approaches [7–9] and predictive models [10–12] have been presented recently for predicting various NM properties and toxicity effects.

The combination of multiple NAMs, both experimental and computational, within an “Integrated Approaches to Testing and Assessment” (IATA) framework will further improve the entire risk evaluation of NMs and accelerate regulatory decision-making procedures [2,5,13]. An IATA scheme for the prediction of the short-term regional lung-deposited dose of inhaled inorganic NMs in humans following acute exposure and the longer-term NM biodistribution after inhalation, has already been presented [14]. Another example of an IATA is the combination of predictions from two or more individual models under a consensus framework. Consensus models combine outputs from several individual models built upon different sets of descriptors and/or machine learning (ML) algorithms, leading to more trustworthy results and enhancing stakeholders’ confidence. In detail, as each individual model covers a specific area of the descriptor/property space, by combining them it is possible to capture a wider range of factors that influence the relationship between the NMs’ independent variables and the endpoint [15–16] and, thus, to approach the problem from different perspectives. Furthermore, by combining different models, it is possible to address the limitations of each model and to achieve more precise predictions (e.g., by avoiding the overfitting phenomenon when small training datasets are involved) [15–16]. Prediction combination can be performed in a regression problem through an arithmetic average or via a weighted average scheme [17]. It has been demonstrated that consensus QSAR models exhibit lower variability than individual models, resulting in more reliable and accurate predictions [18–19]. In the area of nanoinformatics, various consensus approaches have been proposed over the past years for the prediction of different NM endpoints, such as NMs’ cellular uptake [20], zeta potential (ZP) [16], and electrophoretic mobility [21].

The complexity of predictive models requires the development of standardized protocols to ensure their accuracy and robustness. Just as laboratory experiments rely on repeatability and reproducibility to validate results, computational methods require similar validation processes. Special emphasis is given to the predictive accuracy of models. For this purpose, it is sought that nanoinformatics models comply with a set of predefined criteria, often supplemented by statistical methods recommended by the Organisation for Economic Co-operation and Development (OECD) [22] and the European Chemicals Agency (ECHA) [23]. In addition, there is a growing effort from various groups to enhance the transparency and, consequently, the reproducibility of their results by delivering standardized reports along with their models (e.g., QSAR model reporting format (QMRF) [24] and modelling data (MODA) [14,25] reports). By documenting computational steps through the standardized reports, it is possible to deliver reproducible models within and between computational groups, and over time, and to conduct interlaboratory comparisons (ILC) or round-robin (RR) tests on the models and their outputs, like those performed in laboratory settings to validate a new test method or protocol [26–27].

**Figure 1:**
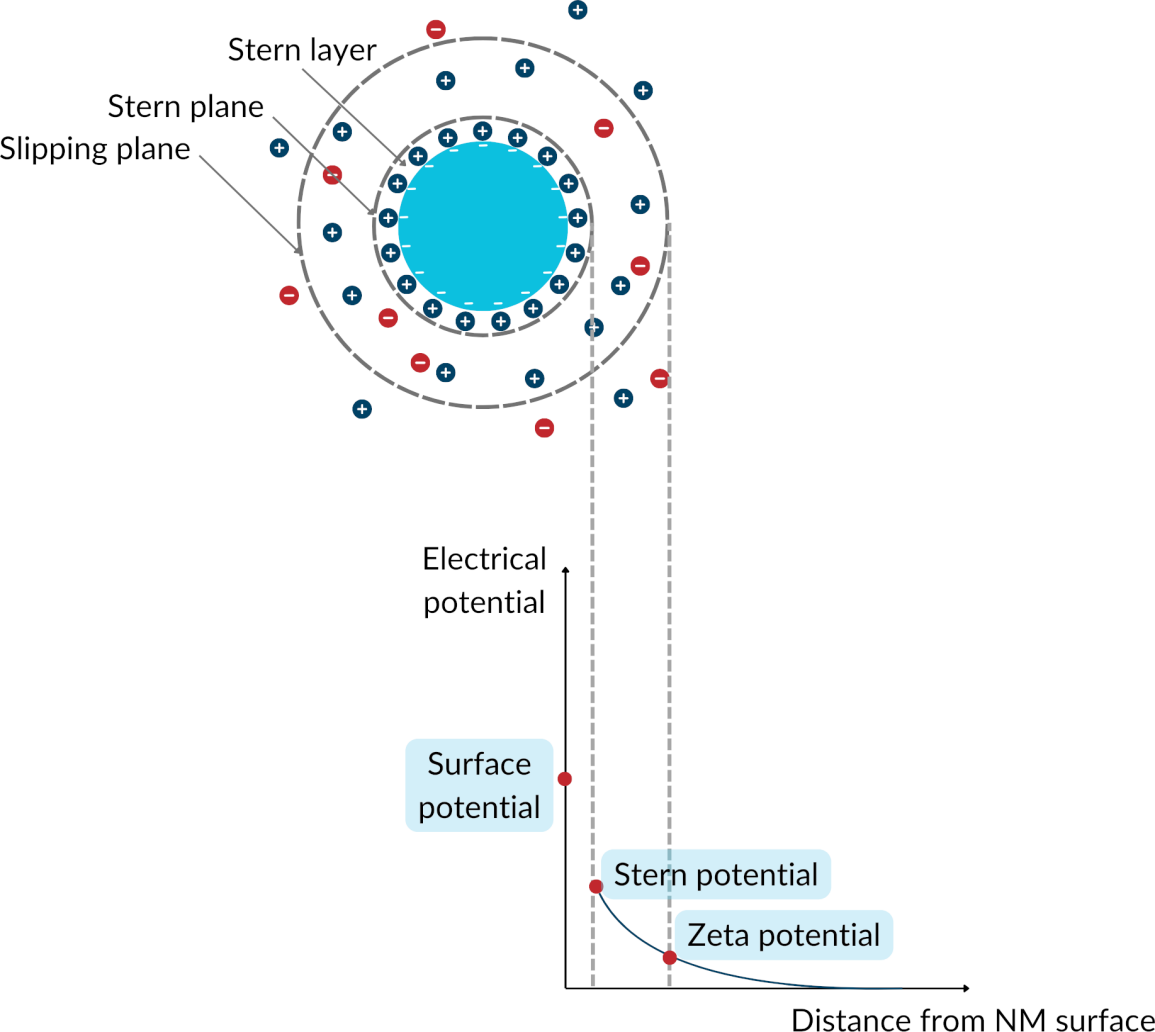
Schematic representation of a negatively charged uncoated spherical NM. The ZP corresponds to the electric charge at the slipping plane.

The computational prediction of the ZP of NMs (Figure 1) has been of high interest in the area of nanoinformatics during the last decade, given the role of surface charge in determining NMs interactions with membranes and in driving toxicity, whereby positively charged particles are generally more toxic than negatively charged particles of similar composition [28–30]. In fact, several in silico models for the ZP have been developed based on different theoretical and experimental descriptors employing a range of approaches, that is, quantitative structure–property/feature relationship (QSPR/QSFR) modelling, read-across, and deep learning models. Mikolajczyk et al. [16] implemented a consensus nano-QSPR scheme for the prediction of the ZP of metal oxide nanoparticles (NPs) based on the size and a quantum mechanical descriptor encoding the energy of the highest occupied molecular orbital per metal atom of 15 metal oxide NPs. Toropov et al. [31] developed, for a set of 15 metal and metal oxide NPs, a QFPR model considering both the NPs’ molecular structure and the experimental conditions, encoded in quasi-SMILES. Furthermore, research has explored the computational assessment of the ZP in media besides water. Wyrzykowska et al. [32] proposed a nano-QSPR model for the prediction of the ZP of 15 NPs in a low-concentration KCl solution considering the NPs’ ZP in water and the periodic number of the NPs metal.

Read-across approaches presented to date include a *k*-nearest neighbours (*k*NN) model developed by Varsou et al. [33] to predict the ZP of 37 metal and metal oxide NPs based on their core type and the NPs main elongation (image descriptor derived from microscopy images). Papadiamantis et al. [34] developed a *k*NN/read-across model for the estimation of the ZP of 69 pristine and aged NPs, considering the size, coating, absolute electronegativity, and periodic table descriptors. Finally, advances of artificial intelligence (AI) have been also considered in the computational assessment of the ZP. Yan et al. [35] employed deep learning techniques and developed a convolutional neural network to predict the ZP of 119 NPs based on their nanostructure images. The abovementioned studies are indicative examples of models that have been used for the computational assessment of NPs ZP. As research progresses, such models are expected to become increasingly sophisticated and accurate, contributing to a deeper understanding of NP behaviour in diverse environments.

The diversity of datasets and endpoints measured is challenging when comparing or combining results between different studies, making it crucial to ensure that data are compatible in terms of metadata (e.g., used experimental protocol). Similarly, models developed using different sets of descriptors need to have a basis for comparison in order to drive regulatory acceptance of models. To address this challenge, under the NanoSolveIT EU project (https://nanosolveit.eu/) the first RR approach in nanoinformatics was implemented, to computationally assess the ZP of NPs. The RR exercise involved four groups (NovaM, NTUA, QSARLab and DTC Lab), from both academia and industry, from four countries (Cyprus, Greece, Poland, and India) who were asked to develop individual models for the prediction of the ZP based on a common dataset of metal and metal oxide-cored NPs. In this way, different descriptors were employed, and various modelling approaches were applied, including QSAR type and read-across models. The developed models were later integrated into a consensus modelling scheme by combining the predictions of the individual models through average and weighted average, to acquire more robust and stable results. While the dataset’s extent and, consequently, the generated models’ applicability domain are rather limited, this initiative underscores the potential of synergistic approaches in the nanoinformatics field. By leveraging the collective knowledge of diverse teams and perspectives, these approaches can effectively assess the properties and toxicity of NPs and democratize decision-making processes in the assessment of NMs’ exposure, hazard, and risk.

## Materials and Methods

### Data overview

A dataset of 71 pristine engineered NMs was explored in silico in order to predict their ZP based on physicochemical and molecular descriptors. The physicochemical characterization of the NMs was performed under the EU-FP7 NanoMILE project (https://cordis.europa.eu/project/id/310451) [36]. From the available descriptors/properties [36], the following four were included in this study because of the completeness of the data (absence of data gaps): the NMs’ core chemistry, coating, morphology, and hydrodynamic diameter measured using dynamic light scattering (DLS). The ZP of the NMs was measured in water (pH 6.5–8.5). To enrich the library of the NMs’ physicochemical properties and increase the amount of available information, the corresponding sphere diameter (the diameter of the sphere with a surface area equal to the area of the NM) was calculated, as well as three molecular descriptors commonly used in nanoinformatics studies [37]. These descriptors were chemical formula-related descriptors, specifically the numbers of metal and oxygen atoms present in the core’s chemical formula and the molecular weight of the core compound.

Finally, the Hamaker constants [38] of the NMs were calculated in vacuum and in water using the NanoSolveIT Hamaker tool (https://hamaker.cloud.nanosolveit.eu/). The Hamaker constant is a material-specific value that quantifies the strength of van der Waals interactions between NPs, depending on the materials and the surrounding medium. A higher (positive) Hamaker constant indicates stronger attractive forces, while a negative value suggests repulsive interactions between the NPs, preventing aggregation or agglomeration. These calculations were performed considering spherical and uncoated NMs. The balance between the Hamaker constants (expressing van der Waals attraction between particles) and the ZP values of particles (expressing their electrostatic repulsion) controls the stability of colloidal dispersions according to the Derjaguin–Landau–Verwey–Overbeek (DLVO) theory [39]. For the computational analysis, the TIP3P force field was employed for water, while the DREIDING force field was used for the NMs. In the case of Zr-doped CeO_2_ NMs (Ce*_x_*Zr*_y_*O_2_), the same density as for pure CeO_2_ NMs was considered to maintain consistency. It should be noted that the different working groups were free to enrich or transform the above-described dataset, as it is explained in the next sections, to cover a wider feature space with each individual model. All the information about the available descriptors is summarised in Table 1. The entire dataset used in the models can be found in the Supporting Information File 1 of this publication.

**Table 1:** Available descriptors in the dataset used to build the individual ZP models (five models from four labs).

Descriptor	Symbol	Unit

chemical formula	CF	—
equivalent sphere diameter	Dsph	nm
shape group	Shape	—
coating	CT	—
hydrodynamic diameter measured by DLS	DLS	nm
molecular weight	MW	g/mol
Hamaker constant of NMs in vacuum	A11	× 10^−20^ J
Hamaker constant of NMs in water	A132	× 10^−20^ J
number of metal atoms	Nmetal	—
number of oxygen atoms	Noxygen	—
sum of ionization potential energy of metals	Metals_SumIP	kJ/mol
a read-across-derived composite function that encodes chemical information from all the selected structural and physicochemical features	RA function	
coefficient of variation of the similarity values of the close source compounds for a particular query compound	CVsim	
total number of atoms in a molecule	Tot num atoms	
weighted standard error of the observed response values of the close source compounds for a particular query compound	SE	
weighted standard deviation of the observed response values of the close source compounds for a particular query compound	SD Activity	
standard deviation of the similarity values of the close source compounds for a particular query compound	SD Similarity	
average similarity values of the positive close source compounds for a particular query compound	Pos.Avg.Sim	
average similarity values of the negative close source compounds for a particular query compound	Neg.Avg.Sim	
the log-transformed hydrodynamic diameter measured by DLS	LOG_DLS	
similarity value of the closest positive source compound	MaxPos	
Banerjee–Roy similarity coefficient 1	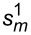	
Banerjee–Roy similarity coefficient 2	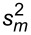	

### Modelling techniques

#### *k*NN/read-across model

The *k*NN/read-across model employs the *k*-nearest neighbours approach, an instance-based method that predicts the endpoint of a sample based on its *k* nearest neighbours in the data space. The proximity between samples is measured using Euclidean distance, which is adjusted slightly for categorical descriptor values using a binary value (0 in the case of same class data points or otherwise 1) [40–41]. The endpoint prediction, in this case the ZP value, is the weighted average of the endpoint values of the *k* closest neighbours, with each neighbour’s weighting factor inversely proportional to its distance from the evaluated sample [33,40].

The *k*NN algorithm can be incorporated into the general NMs read-across framework because it relies on the similarity of neighbouring NMs to estimate the endpoint of interest. Specifically, by identifying and analysing the resulting groupings, it is possible to map the prediction space into distinct clusters of *k* neighbours that can subsequently be explored to identify patterns and similarities within the neighbourhood space, in accordance with the ECHA’s read-across framework. The Enalos*k*NN functionality offers the advantage of not only delivering predictive results but also identifying the specific neighbours and their Euclidean distances, as well as enabling visualization of the overall prediction space [33–34].

#### Random forest regression model

Random forest regressor is an ensemble learning, tree-based method. It combines multiple decision tree predictors to create a more robust and accurate prediction, which individual trees cannot always provide. This algorithm constructs a forest of independent trees. Each tree is being trained on a random subset of data and features. The regressor’s output is calculated based on the average predictions from all individual trees. Some benefits of this algorithm besides its robustness include resistance to overfitting and the ability to process datasets with numerous variables without the need of feature scaling [42]. This algorithm was implemented in Python, using scikit-learn package, a widely used library for ML models.

#### Adaboost regression model

The development of the ZP QSPR model involved the utilization of the Adaptive Boosting (AdaBoost) ML methodology, implemented through Python 3.8.8 and the scikit-learn library (version 0.24.1). AdaBoost represents an early instance of leveraging boosting algorithms to address complex problem types within the domain of ML [43]. Like its counterpart, the random forest algorithm, AdaBoost employs a multitude of elementary classifiers to enhance the model’s predictive ability. In brief, the AdaBoost model comprises an ensemble of multiple “weak” estimators, such as decision trees, each possessing modest individual predictive prowess. However, when integrated into an ensemble, they collectively augment the predictive efficiency of the model. A notable distinction between the random forest algorithm and AdaBoost lies in their operational frameworks. In the random forest, individual estimators function independently of each other, operating in parallel. In contrast, in AdaBoost, the prediction process within the ensemble unfolds sequentially, with each subsequent estimator’s outcome influenced by its predecessor.

#### Stacked PLS and MLP q-RASPR models

The q-RASPR approach, combining read-across and QSPR, has been recently introduced and applied to the prediction of NM cytotoxicity [44], power conversion efficiency of organic dyes in dye-sensitized solar cells [45–46], detonation heat for nitrogen containing compounds [47], and to the prediction of surface area of perovskite materials [48]. Both the QSPR and read-across approaches are extensively used for data gap filling (predicting activity/property/toxicity values of compounds devoid of experimentally derived endpoint values). Recently, Luechtefeld et al. [49] introduced the concept of classification-based read-across structure–activity relationship (RASAR) by combining the concepts of read-across and QSAR using ML algorithms. Banerjee and Roy [50] merged chemical read-across and regression-based QSAR into quantitative RASAR (q-RASAR). Several ML models can be applied including partial least squares (PLS), linear support vector regression (LSVR), random forest regression, Adaboost, multiple layer perceptron (MLP) regression, and *k*NN regression. This study reports the first application of q-RASPR in a stacked modelling framework.

Apart from the supplied structural and physicochemical information of the engineered NMs, we have computed descriptors based on the periodic table using the tool Elemental Descriptor Calculator (https://sites.google.com/jadavpuruniversity.in/dtc-lab-software/other-dtc-lab-tools). The complete descriptor pool underwent feature selection using stepwise selection and a genetic algorithm to obtain a reduced descriptor pool consisting of 72 descriptors. A grid search/best subset selection was applied to this reduced descriptor pool to obtain a combination of ten different QSPR descriptors. Additionally, log-transformed hydrodynamic diameter (LOG_DLS) was taken as an additional descriptor. These eleven QSPR descriptors were used to define similarity among the source and query compounds, which is an integral part of the computation of the RASPR descriptors using the tool RASAR-Desc-Calc-v3.0.2 available from https://sites.google.com/jadavpuruniversity.in/dtc-lab-software/home. This tool uses three different algorithms for computing similarity, that is, Euclidean distance-based, Gaussian kernel similarity-based and Laplacian kernel similarity-based. The selection of the best similarity measure and the optimization of the associated hyperparameters were performed by dividing the training set into calibration and validation sets, which were supplied as inputs for the tool Auto_RA_Optimizer-v1.0 available from https://sites.google.com/jadavpuruniversity.in/dtc-lab-software/home. The combination of hyperparameters that generated the best predictions for the validation set was selected as the optimized hyperparameter setting and used to compute the RASPR descriptors for the training and test sets. Clubbing of the initially selected eleven QSPR descriptors with the RASPR descriptors was performed, a process known as data fusion [51]. This complete data pool underwent feature selection to generate four different MLR q-RASPR models. The predictions from these models were generated for both the training and test sets since these predictive values will serve as descriptors for the final stacking regressors. Finally, PLS and MLP modelling algorithms were employed as the final stacking regressors, where the optimized settings of the hyperparameters were obtained by grid search on the cross-validation statistics.

### Consensus modelling

The meta-modelling approach allows one to use the output of one modelling approach as an input to another or the use of a few models/algorithms in parallel or in sequence, allowing for the strengths of individual models to be combined and their limitations to be circumvented [15,52]. Consensus modelling is based on the parallel approach where multiple ML algorithms are used to investigate the available dataset and to find relationships between the considered NMs’ features and the physicochemical descriptors or biological activity of interest. Each ML algorithm has its strengths and weaknesses; thus, there is no universal solution for modelling regression or classification cases. The choice of the adequate ML method depends on the problem to be solved and the available data, and in some cases multiple methods are employed to decide which one works best for each case [53–54]. Depending on the amount of available data, different methods may be applied. In general, support vector machines, decision trees, random forests, and neural networks are methods good in generalisation of trends or behaviours and can lead to accurate predictions. However, in cases of small datasets, the same ML methods may lead to the overfitting and low predictivity of the model for untested samples. The idea of consensus modelling by combining a set of diverse algorithms for the prediction endpoint of interest is an efficacious manner to achieve reliable results of data-driven analysis. However, this approach is also open to criticism that it is even more “black box” than the individual models; thus, even more care needs to be taken to fully document the predictive models with their QMRFs reports and to fully describe the underpinning datasets.

Here, a consensus strategy was employed in addition to the individually developed models, based on the combination of the predictions from the initial models generated by the four groups NovaM, NTUA, QSARLab, and DTC Lab. Two techniques were used to derive consensus predictions, namely, the simple average of the predictions of the individual models and the weighted average of the original predictions. Simple averaging combines the predictions of all individual models equally, while weighted averaging assigns more weight to models with higher individual performance. This combination aims to leverage the strengths of each model, reducing individual biases and enhancing overall prediction accuracy.

### Validation

In line with the OECD QSAR model validation principles [22,55], all models presented in this work were validated externally using the exact same training and test sets, which were produced by randomly dividing the original dataset using a ratio of 0.75:0.25. The training subset was used each time to calculate and adjust the model parameters, whereas the test subset was not involved in model development, and it was used as an external validation set to assess the model’s generalization on new (previously unseen) data, which is crucial for its practical application in regulatory settings.

According to the OECD’s fourth principle [22], statistical model validation is indispensable for assessing a model’s performance. To quantify the model’s accuracy, appropriate “fitness” metrics were employed, ensuring that the models’ predictions closely align with their actual values. This validation process helped to prevent underfitting and overfitting phenomena. Upon training, the models generated endpoint predictions for both the training and test subsets. The training subset predictions served to evaluate each model’s goodness-of-fit, while predictions on the test subset assessed the model’s predictability, for example, its ability to generalize well to new data [22]. The statistical criteria used to evaluate model performance are outlined below. These metrics collectively provide a comprehensive assessment of model accuracy and reliability.

The mean absolute error (MAE, Equation 1) and the root mean squared error (RMSE, Equation 2) were used to evaluate the accuracy of the models applied on both train and test sets. MAE measures the average magnitude of errors in predictions, while RMSE provides a quadratic scoring rule that gives higher weight to larger errors. When these indexes are used simultaneously, they permit a complete and thorough validation of prediction accuracy, regardless of the training and test endpoint values’ distribution level. MAE and RMSE values closer to 0, correspond to more reliable models.


[1] $$\text{MAE}=\frac{1}{N}\sum_{i=1}^{N}\left|y_{i}-{\hat{y}}_{l}\right|$$

[2] $$\text{RMSE}=\sqrt{\frac{1}{N}\sum_{i=1}^{N}{\left(y_{i}-{\hat{y}}_{l}\right)}^{2}}$$

where *N* is the number of samples, and *y_i_* and 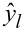 are the actual and predicted endpoint values of the *i*-th sample, respectively.

The quality-of-fit between the predicted and experimental values of the training and test sets was expressed by the coefficient of determination (*R*^2^, Equation 3), which indicates the proportion of variance in the dependent variable that is predictable from the independent variables. *R*^2^ values closer to 1, correspond to models that fit the dataset better.


[3] $$R^{2}=1-\frac{\sum_{i=1}^{N}{\left(y_{i}-{\hat{y}}_{l}\right)}^{2}}{\sum_{i=1}^{N}{\left(y_{i}-\bar{y}\right)}^{2}}$$

where *N* is the number of samples, *y_i_* and 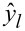 are the actual and predicted endpoint values of the *i*-th sample, respectively, and 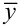 is the average value of the experimental endpoint values.

To quantify the credibility of predictions on new data (including the test set), the external explained variance [22] is used (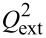 or 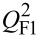, Equation 4), which compares the predictions for the test set samples with their actual endpoint values. 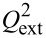 values closer to 1, correspond to models with higher predictive power.


[4] $$Q_{\text{ext}}^{2}=1-\frac{\sum_{i=1}^{N}{\left(y_{i}-{\hat{y}}_{l}\right)}^{2}}{\sum_{i=1}^{N}{\left(y_{i}-{\bar{y}}_{\text{tr}}\right)}^{2}}$$

where *N* is the number of test samples, *y_i_* and 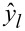 are the actual and predicted endpoint values of the *i*-th test sample, respectively, and 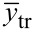, is the averaged value of the experimental endpoints of the training set.

Another variant of the external explained variance is 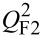 (Equation 5) which uses the averaged value of the experimental endpoints of the test set (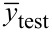).


[5] $$Q_{\text{F}2}^{2}=1-\frac{\sum_{i=1}^{N}{\left(y_{i}-{\hat{y}}_{l}\right)}^{2}}{\sum_{i=1}^{N}{\left(y_{i}-{\bar{y}}_{\text{test}}\right)}^{2}}$$

The produced models were validated internally by employing leave-one-out (LOO) cross-validation on the training set, to ensure that the model is robust and no single data point is actually responsible for the enhanced quality of fit. The performance in LOO cross-validation was assessed by calculating 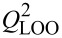 (leave-one-out *Q*^2^), a form of cross-validated *R*^2^ of the predictions (Equation 6) [56].


[6] $$Q_{\text{LOO}}^{2}=1-\frac{\sum_{i=1}^{N}{\left(y_{i}-{\hat{y}}_{l}\right)}^{2}}{\sum_{i=1}^{N}{\left(y_{i}-\bar{y}\right)}^{2}}$$

where *N* is the number of training samples, *y_i_* and 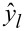, are the actual and predicted from LOO cross-validation endpoint values of the *i*-th sample, respectively, and 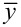 is the average value of the experimental training endpoint values.

Finally, the quality-of-fit and the predictive ability of the models is assessed using the statistical metrics proposed by Golbraikh and Tropsha [57–58] (Equations 7–11, including 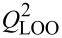, Equation 6) on the test set. According to Golbraikh and Tropsha [57,59–60] a regression model is considered predictive if all of the conditions presented in Table 2 are satisfied.


[7] $$r^{2}={\left(\frac{\sum_{i=1}^{N}\left(y_{i}-\bar{y}\right)\left({\hat{y}}_{l}-{\bar{\hat{y}}}_{l}\right)}{\sqrt{\sum_{i=1}^{N}{\left(y_{i}-\bar{y}\right)}^{2}\sum_{i=1}^{N}{\left({\hat{y}}_{l}-{\bar{\hat{y}}}_{l}\right)}^{2}}}\right)}^{2}$$

[8]

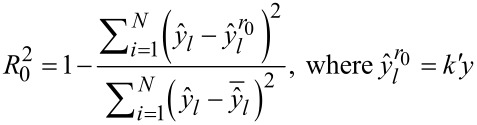




[9]

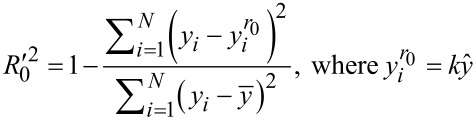




[10] $$k=\frac{\sum_{i=1}^{N}y_{i}{\hat{y}}_{l}}{\sum_{i=1}^{N}{\hat{y}}_{l}^{2}}$$

[11] $$k^{\prime }=\frac{\sum_{i=1}^{N}y_{i}{\hat{y}}_{l}}{\sum_{i=1}^{N}y_{i}^{2}}$$

where *N* is the number of samples, *y_i_* and 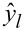 are the actual and predicted endpoint values of the *i*-th sample, respectively, and 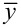 and 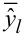 are the average endpoint values of the experimental and predicted values, respectively.

**Table 2:** Model acceptability criteria as defined by Golbraikh and Tropsha [57,59–60].

Statistic	Rule

*r* ^2^	>0.6
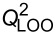	>0.5
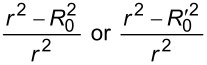	<0.1
*k* or *k*’	∈[0.85,1.15]
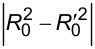	<0.3

### Applicability domain

To ensure the robustness and reliability of predictive models, particularly adhering to the OECD guidelines, defining the applicability domain (AD) is crucial. The AD refers to the specific subset of the overall data space where a model can make reliable predictions through interpolation. When the model encounters data points beyond this designated domain, those predictions should be flagged as unreliable because of their extrapolation-based nature, which inherently carries more uncertainty than interpolation [22].

In the present study, the leverage method [61] was employed to assess the prediction reliability. This was done to empower users to apply the models with greater confidence to external datasets and real-world scenarios while having, at the same, time a clear understanding of their optimal operating parameters. The leverage method measures the similarity between the query samples and the training set using the leverage values, *h*, which are essentially the diagonal elements of the Hat matrix [61–62] (Equation 12). These values quantify the distance of each query sample from the centroid of the training set [61], taking into account the descriptor values employed in model development. The AD boundaries are determined by a predetermined threshold leverage value h* (Equation 13). A test prediction is deemed reliable if its corresponding leverage value falls below this threshold (*h* < h*).


[12] $$H=X{\left(X^{T}X\right)}^{-1}X^{T}$$

[13] $$h^{*}=3\times \frac{p}{N}$$

where *X* is the table containing the descriptor matrix, *p* is the number of descriptors used in the model [60–61], and *N* is the number of samples in the training dataset.

## Results and Discussion

In the next paragraphs the five developed individual models are briefly described. To ensure fair comparison, all models were trained and tested on identical subsets of the data. More information can be found in the respective QMRF reports, provided as Supporting Information Files 2–5 to this publication.

### *k*NN/read-across model

#### Data preprocessing

Initially, the z-score normalisation method was employed to standardise the descriptors in the training set (53 NMs), ensuring their equal contribution to the model. Each descriptor was adjusted to have a mean of zero and a standard deviation of one [24]. Next, the identical normalisation parameters were applied to the descriptors in the test set (18 NMs). To identify the most relevant parameters, eliminate noise, and avoid overfitting, the *BestFirst* method with the *CfsSubset* evaluator were employed [40]. Four descriptors were selected to use in the model (see below Table 15), that is, the NMs’ coating, their equivalent sphere diameter, their hydrodynamic diameter, and the number of oxygen atoms present in the core’s chemical formula. To enhance the model’s performance and interpretability, the Hamaker constant of the NMs calculated in water and the shape group were added to the subset of the selected descriptors. All analysis steps were performed in Isalos Analytics Platform [63].

#### Model development and validation

The *k*NN algorithm with a value of *k =* 7 was selected to perform a read-across assessment of the dataset. Similarly to the preprocessing steps, modelling was implemented in Isalos Analytics Platform using the Enalos+ tools and especially the Enalos*k*NN function [24]. This function identifies the neighbouring training samples for each test NM alongside the predicted values, facilitating a deeper understanding of the results in terms of NM grouping and providing insights into the overall samples space. The model was validated following the OECD principles [22] to ensure robust and reliable predictive modelling. The key statistical metrics of internal (training set) and external (test set) validation are presented in Table 3. The Y-randomization test [24] was also performed ten times, giving RMSE values on the test set in the range of 23.1–43.4, confirming that the predictions were not a coincidental outcome. In Table 4 the results of the Golbraikh and Tropsha [57,59–60] test for the *k*NN/read-cross model are presented.

**Table 3:** Internal (training set) and external (test set) validation statistics of the *k*NN/read-across model.

	Training set	Test set

MAE	0.29	7.81
RMSE	0.54	9.71
*R* ^2^	0.99	0.88
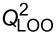	0.62	—
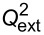	—	0.88

**Table 4:** Golbraikh and Tropsha [57,59–60] test results for the *k*NN/read-cross model.

Criterion	Assessment	Result

*r*^2^ > 0.6	pass	0.894
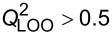	pass	0.622
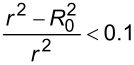	pass	0.001
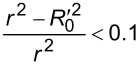	pass	0.002
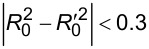	pass	0.001
0.85 < *k* < 1.15	pass	0.883
0.85 < *k*′ < 1.16	pass	1.012

#### Applicability domain

The area of reliable predictions for this model was defined using the leverage method. The leverage threshold was calculated based on the training NMs subset and set to 0.226 (Equation 13). The test NM samples had values within the range of 0.031 to 0.191, indicating that their predictions were reliable except the one NM sample whose leverage value was equal to 0.859.

### Random forest regression model

#### Data preprocessing

To facilitate data analysis, the unique string feature names of the chemical formula descriptors were converted into a binary variable. For this purpose, metal oxides (e.g., CeO_2_ and CuO) were represented as 0 and metals (e.g., Ag, Au, and Cu) were represented as 1. For the shape group descriptor, the string names “Spherical”, “Square Plates” and “Rod” were one-hot encoded. Lastly, out of 22 unique coatings, five categories were created (sodium citrate, ʟ-arginine, PVP, uncoated, and “other”) and were one-hot-encoded as well. This conversion ensured consistency and uniformity in data representation, making it easier to handle and analyse the data effectively. Next, Pearson’s correlation value was computed for each pair of descriptors. The two Hamaker constants (in water and in vacuum) had a correlation value of 0.97, indicating that these two features were linearly dependent. Thus, to avoid introducing redundancy and potential issues in the ML model, the Hamaker constant in vacuum was removed.

#### Model development and validation

A random forest regressor was trained on the training set using Jupyter notebook and the scikit-learn ML package. To optimize the model’s performance, the grid search algorithm was implemented to tune the model using the 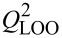 metric for internal validation. To further enhance the predictive power of the model, recursive feature elimination (RFE) was employed to identify and eliminate descriptors that contributed minimally to the model’s prediction accuracy. After this extensive parameter tuning, the optimal model was identified (128 estimators, maximum depth of five and random state equal to 42) as well as the optimal features (DLS, coating, equivalent sphere diameter, and MW) achieving 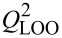 = 0.611 and *R*^2^ = 0.957 on the training set and *R*^2^ = 0.941 on the test set. The key model statistics are presented in Table 5, and the results of the Golbraikh and Tropsha [57,59–60] tests for the random forest regression model are presented in Table 6.

**Table 5:** Internal (training set) and external (test set) validation statistics of the random forest regression model.

	Training set	Test set

MAE	4.43	5.43
RMSE	6.76	6.73
*R* ^2^	0.96	0.94
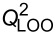	0.61	—
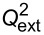	—	0.94

**Table 6:** Golbraikh and Tropsha [57,59–60] test results for the random forest regression model.

Criterion	Assessment	Result

*r*^2^ > 0.6	pass	0.941
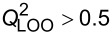	pass	0.611
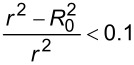	pass	0.0003
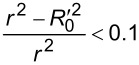	pass	0.0004
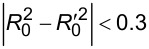	pass	0.0002
0.85 < *k* < 1.15	pass	1.006
0.85 < *k*’ < 1.16	pass	0.936

#### Applicability domain

For the applicability domain, leverage was used to see if the NMs were within the area of reliable predictions. The leverage threshold, calculated on the training set, was set to h^*^ = 0.509. In the training set, one compound had *h* = 0.54, and in the test set one NM had *h* = 0.94. Thus, predictions of those two NMs are not considered reliable.

### AdaBoost regression model

#### Data preprocessing

The initial phase of feature selection involved categorizing descriptors into those with continuous numerical values (e.g., hydrodynamic diameter) and those with qualitative or “descriptive” details (e.g., chemical formula, shape group, and coating). The collection of descriptors characterised by continuous numerical values was subsequently delineated as the “continuous set” for clarity purposes.

The transformation of the descriptive category of descriptors into binary representations was carried out to facilitate the inclusion of these qualitative descriptors in ML algorithms. Binary encoding allows for the representation of categorical variables as binary vectors, where each category variant is encoded as 0 or 1, respectively. This transformation is essential because many ML algorithms require input data to be in numerical form. By converting descriptive features into binary format using the OneHotEncoder from the scikit-learn library, we ensure compatibility with these algorithms while retaining the inherent information encoded within the descriptors. This obtained set is denoted as the “binary set” including the “Chemical formula”, “Shape group”, and “Coating” descriptors. Continuous descriptors were standardized using z-score normalization to ensure equal contribution to the model, using the StandardScaler module from the scikit-learn library. Next, the two sets of data, that is, the standardised continuous set and the binary set, were merged into a unified dataset that enabled us to explore relationships between different types of descriptors and their collective influence on the NMs ZP.

During the initial modelling phase, the AdaBoost algorithm, integrated within the scikit-learn library, was utilized to analyse the comprehensive dataset comprising all descriptors. The primary objective of this approach was to identify the descriptors possessing the highest degree of influence for subsequent modelling tasks. Additionally, pivotal parameters crucial for refining the model’s performance, including “n_estimators”, “random_state”, “learning_rate” were carefully selected during this stage based on GridSearch algorithm for tuning hyperparameters of the model [64]. Detailed insights into these parameters can be accessed via the documentation provided on the official scikit-learn website [65].

After the evaluation of the model’s feature importance, delineated in the preceding stage, five descriptors emerged as the most significant for the ZP prediction, namely, DLS, Dsph, A11, MW, and CT [encoded as 0 = coated and 1 = uncoated]. Each descriptor offers crucial insights into different aspects of the NMs’ composition, structure, and behaviour, thereby serving as vital predictors for the model’s predictive accuracy and interpretability.

#### Model development and validation

The selected descriptors were employed in the training of the final model, which adhered to the methodological framework outlined above. This model was instantiated with specific parameter settings, as elucidated in the previous point, where AdaBoost was configured with parameters: n_estimators = 9, random_state = 786, and learning_rate = 0.997. A number of estimators (n_estimators) were found to enhance the model’s predictive power, while the specific random_state ensures reproducibility of results. Additionally, the learning rate was carefully tuned to strike a balance between model complexity and generalization ability, ultimately resulting in a well-performing model for the given task.

The model validation statistics and the results of the Golbraikh and Tropsha [57,59–60] test are presented in Table 7 and Table 8, respectively.

**Table 7:** Internal (training set) and external (test set) validation statistics of the AdaBoost regression model.

	Training set	Test set

MAE	7.44	8.95
RMSE	9.98	9.91
*R* ^2^	0.91	0.87
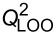	0.54	–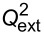	–	0.88

**Table 8:** Golbraikh and Tropsha [57,59–60] test results for the AdaBoost regression model.

Criterion	Assessment	Result

*r*^2^ > 0.6	pass	0.906
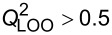	pass	0.539
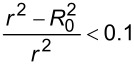	pass	0.027
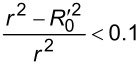	pass	0.028
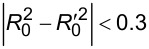	pass	0
0.85 < *k* < 1.15	pass	0.906
0.85 < *k*’ < 1.16	pass	0.974

### Stacked PLS and MLP q-RASPR models

#### Data preprocessing

First- and second-generation periodic table descriptors were calculated as described by Roy and Roy [66]. Some descriptors were also calculated using elemental descriptors calculator software (https://sites.google.com/jadavpuruniversity.in/dtc-lab-software/other-dtc-lab-tools?authuser=0). Basic information about the metals has been taken directly from the periodic table to calculate descriptors for the reported metal oxide NMs.

Additional information on physicochemical features such as coating, shape group, DLS (hydrodynamic diameter) [nm], Hamaker (self/vacuum) A11 [× 10^−20^ J], Hamaker (self/water) A132 [× 10^−20^ J] were also included for modelling purposes. The selected QSPR descriptors (vide infra) were used to compute the RASPR descriptors using the tool RASAR-Desc-Calc-v3.0.2 (https://sites.google.com/jadavpuruniversity.in/dtc-lab-software/home#h.x3k58bv4frb9) after optimization of the associated read-across-based hyperparameters [67–68].

#### Model development and validation

The model development was performed following the basic steps for the generation of the MLR model using the best subset selection (BSS) method. The data division was kept identical to the data partitioning used in the rest of the models to have a clear comparison of results. Further, Stepwise Selection (using *F*-value as the fitness function) and Genetic Algorithm (GA) (using MAE_train_ as the fitness function) were implemented for feature selection followed by the BSS method to select the best model based on the quality and prediction performance.

**Initially selected QSAR descriptors (obtained by the grid search algorithm).** Ten descriptors (from a total of 72 descriptors) were obtained after Stepwise Selection, GA, and BSS. These are Hamaker (self/water), amount of Ce, amount of Zr, rod (shape), coating, the total number of atoms, tot_metal_alpha, Metals_SumIP, X_ActivM, and Valence electron potential.

Additionally, we performed a correlation analysis of the descriptor DLS (hydrodynamic diameter) and found that it had a significant correlation with the training set response, except for four data points. This was because, for these compounds, the values of DLS were significantly higher than the rest of the training data points, therefore hindering linear correlation. Thus, we have converted the DLS descriptor to the corresponding log unit, added this feature to the initially selected ten features, and considered it for model development. Therefore, we have proceeded toward further modelling analysis using eleven QSAR descriptors.

**RASPR descriptor computation.** Using these selected features, the read-across structure–property relationship (RASPR) descriptors [67] for the training and test sets were computed using the tool RASAR-Desc-Calc-v3.0.2, freely available from the DTC Lab tools supplementary site (https://sites.google.com/jadavpuruniversity.in/dtc-lab-software/home#h.x3k58bv4frb9). The corresponding hyperparameter (similarity based on Euclidean distance with the number of close source compounds equal to 5) settings were obtained from the optimized read-across-based predictions for the validation set, using the calibration set as the source set (the calibration and validation sets were obtained by the division of the training compounds). This read-across hyperparameter optimization was done using the tool Auto_RA_Optimizer-v1.0, freely available from the DTC Lab tools supplementary site (https://sites.google.com/jadavpuruniversity.in/dtc-lab-software/home#h.ucbojxjcke1c).

The 18 different RASPR descriptors computed were fused with the initially selected QSPR descriptors to generate complete descriptor pools for the training and test sets, a process termed Data Fusion [51]. This pool was subjected to feature selection using a grid search algorithm.

**Figure 2:**
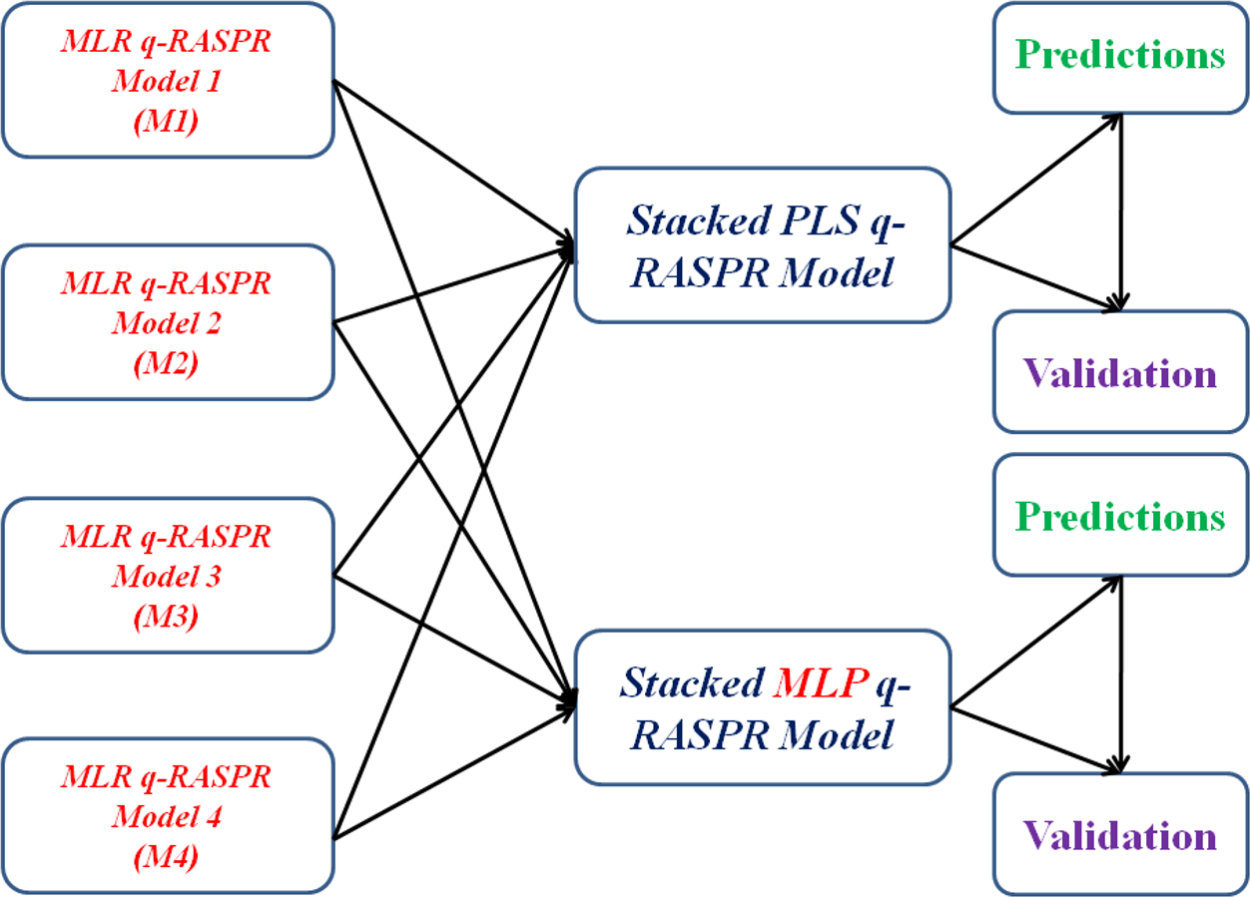
Schematic workflow for the development of the stacked PLS and MLP q-RASPR models.

From the results of the grid search, four different MLR q-RASPR models were developed. The corresponding descriptors associated with the four different MLR models have been tabulated in Table 9, while the internal and external validation metrics of these individual models have been reported in Table 10. Their individual predictions were used to perform stacking using a PLS algorithm (using the optimized number of latent variables (LVs) based on LOO cross-validation) as the final regressor (Figure 2), the results of which have been reported in Table 11 and Table 12.

**Table 9:** Descriptor combination of the MLR q-RASPR models.

Models	Desc1	Desc2	Desc3	Desc4	Desc5	Desc6

M1	Metals_SumIP	RA function	CVsim	Pos.Avg.Sim	Neg.Avg.Sim	s_m_^1^
M2	LOG_DLS	SE	SD Similarity	Pos.Avg.Sim	Neg.Avg.Sim	s_m_^2^
M3	Tot num atoms	LOG_DLS	SD Activity	MaxPos	Neg.Avg.Sim	s_m_^1^
M4	LOG_DLS	SD Activity	MaxPos	SD Similarity	Neg.Avg.Sim	s_m_^1^

**Table 10:** Internal (training set) and external (test set) validation statistics of the individual MLR q-RASPR models.

Models	Training set		Test set				

	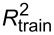	MAE_train_	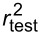	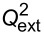	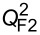	MAE_test_	RMSEP

M1	0.629	14.837	0.972	0.974	0.972	3.671	4.605
M2	0.694	11.937	0.930	0.881	0.873	7.539	9.833
M3	0.661	14.082	0.959	0.955	0.952	4.969	6.068
M4	0.652	13.712	0.942	0.944	0.941	5.276	6.730

**Table 11:** Internal (training set) and external (test set) validation statistics of the stacked PLS q-RASPR regression models.^a^

Stacked PLS q-RASPR (training set statistics)	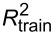	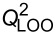	MAR_train_	MAE_LOO–CV_	RMSEC
	0.681	0.657	13.255	13.766	18.417

Stacked PLS q-RASPR (test set statistics)	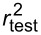	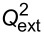	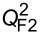	MAE_test_	RMSEP
	0.960	0.951	0.948	4.402	6.320

^a^The optimized hyperparameter setting for the Stacked PLS q-RASPR model is LV = 1.

**Table 12:** Golbraikh and Tropsha [57,59–60] test results for the stacked PLS q-RASPR model.

Criterion	Assessment	Result

*r*^2^ > 0.6	pass	0.960
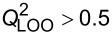	pass	0.657
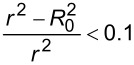	pass	0.001
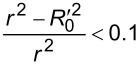	pass	0.001
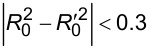	pass	0
0.85 < *k* < 1.15	pass	0.902
0.85 < *k*’ < 1.16	pass	1.063

Apart from PLS, we have also used a MLP model as the final regressor (Figure 2) after optimization of the hyperparameters by the GridSearchCV approach. The validation statistics are presented in Table 13 and Table 14.

**Table 13:** Internal (training set) and external (test set) validation statistics of the stacked MLP q-RASPR regression models.^a^

Stacked MLP q-RASPR (training set statistics)	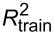	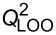	MAE_train_	MAE_LOO–CV_	RMSEC
	0.695	0.645	12.952	13.957	18.015

Stacked MLP q-RASPR (test set statistics)	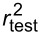	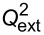	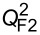	MAE_test_	RMSEP
	0.961	0.963	0.960	4.038	5.500

^a^The optimized hyperparameter settings for the Stacked MLP q-RASPR model are activation = “logistic”, alpha = 1, learning_rate_init = 0.01, max_iter = 1000, random_state = 0, and solver = “lbfgs”.

**Table 14:** Golbraikh and Tropsha [57,59–60] test results for the stacked MLP q-RASPR model.

Criterion	Assessment	Result

*r*^2^ > 0.6	pass	0.961
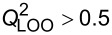	pass	0.645
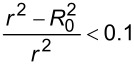	pass	0
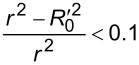	pass	0
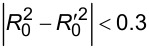	pass	0
0.85 < *k* < 1.15	pass	0.991
0.85 < *k*’ < 1.16	pass	0.970

### Consensus models

The efficacy of the two proposed consensus approaches based on averaging with equal weights or on weighted calculations (Equation 14), was assessed through comparing prediction results for the test set, where the same training and test sets were used for the five individual models, but using different sets of descriptors (Table 15). The consensus predictions using the averaging scheme were derived using the test set predictions of the five individual models with equal weights in the calculation of the final predictions. In this manner, averaged statistical parameters were calculated (Table 16).

**Table 15:** Selected descriptors per model.

*k*NN/read-across	Random forest regression	Adaboost regression	Stacked PLS – q-RASPR	Stacked MLP – q-RASPR

Dsph	Dsph	Dsph		
CT	CT [unique integers]	CT [binary]		
DLS	DLS	DLS		
	MW	MW		
A132				
		A11		
Noxygen				
Shape				
				
			Ypred(M1)^a^	Ypred(M1)
			Ypred(M2)^b^	Ypred(M2)
			Ypred(M3)^c^	Ypred(M3)
			Ypred(M4)^d^	Ypred(M4)

^a^Predicted values from the individual q-RASPR model M1. ^b^Predicted values from the individual q-RASPR model M2. ^c^Predicted values from the individual q-RASPR model M3. ^d^Predicted values from the individual q-RASPR model M4.

**Table 16:** Accuracy statistics on the test set for the five independent models and the two consensus models.

Statistic	kNN/read-across	Random forest regression	Adaboost regression	Stacked PLS – q-RASPR	Stacked MLP – q-RASPR	Consensus average	Consensus weighted average

*R* ^2^	0.88	0.94	0.87	0.95	0.96	0.97	0.97
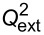	0.88	0.94	0.88	0.95	0.96	0.97	0.97
MAE	7.81	5.43	8.95	4.40	4.04	4.01	4.35
RMSE	9.71	6.73	9.91	6.32	5.50	4.86	5.03

In the weighted average consensus scheme, the weights were calculated based on the coefficient of determination 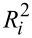 values of the five models on the training set as follows:


[14] $$\hat{y}=\frac{R_{i}^{2}}{\sum R_{i}^{2}}{\hat{y}}_{i}$$

**Figure 3:**
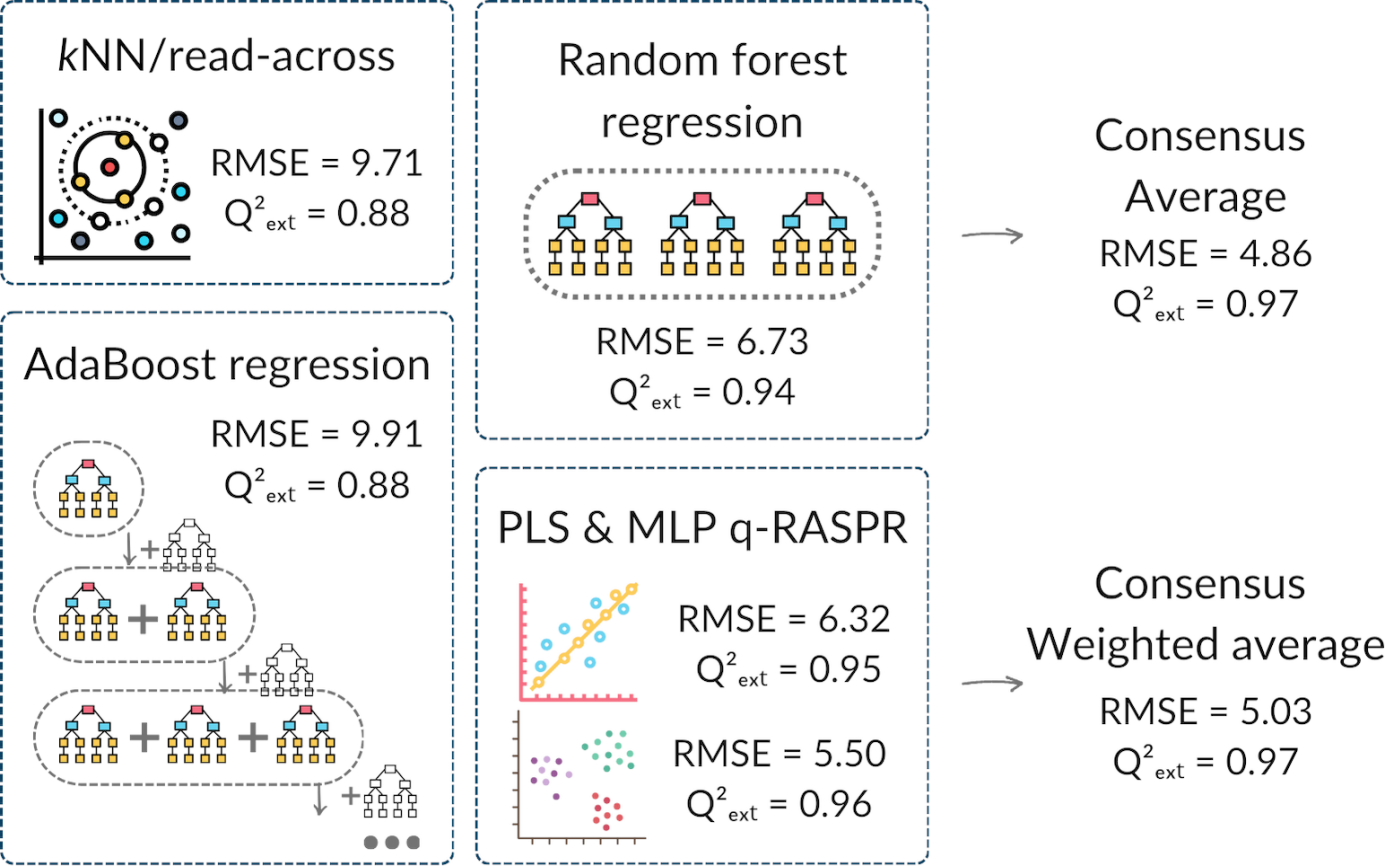
Schematic representation of the individual and consensus models for the RR exercise. The five models were developed independently by four different groups and were later combined into a simple average and a weighted average scheme (consensus models). The consensus models present improved predictive accuracy compared to the individual initial models.

The consensus predictions on the test set were validated for their reliability using the same statistical metrics and the results are presented in Table 16. The obtained results for both consensus approaches are much better than those of the individual models, that is, *R*^2^ and 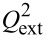 are closer to 1, while RMSE is closer to 0. This confirms the usefulness of integrating diverse ML approaches for more reliable results. The results of the RR exercise presented herein (Figure 3) show that the diverse ML modelling techniques like read-across and QSPR can be applied, and diverse sets of descriptors can be used, to calculate key nanomaterials properties. Nevertheless, the best results can be achieved through the combination of various solutions via consensus modelling, which is recommended for enhanced accuracy and reliability of the prediction of the most important nanomaterials endpoints.

## Conclusion

In this collaborative work we have implemented a round-robin (RR) test focused on the creation of two consensus models for the prediction of the zeta potential (ZP) of metal and metal oxide NMs in aqueous environments. Four distinguished nanoinformatics groups participated in this exercise, each developing their own models based on a shared NMs dataset. The models developed as part of the RR test included (i) a *k*-nearest neighbours algorithm coupled with a read-across approach, enabling a nuanced exploration of the similarity space among the materials being studied, (ii) a random forest model, and (iii) an AdaBoost regression model, both of which stand out for their speed and computational efficiency. Last, two quantitative read-across structure-property relationship (q-RASPR) models were included that combine the advantages of read-across and QSAR approaches. Each of these individual models has been rigorously tested and validated, adhering to the OECD principles to ensure their reliability and predictive accuracy, as described herein.

The key innovation lies in the next step, that is, in the combination of these individually potent models into a consensus framework. We created two different ensemble models for this purpose. The first ensemble model was straightforward; it averaged the predictions coming from all four individual models. This averaging method effectively pooled the strengths of the individual models to produce a more robust predictive output. The second ensemble model took a more nuanced approach, utilising a weighted average scheme. Both consensus models demonstrated an improvement in predictive accuracy compared to their individual components. Moreover, by pooling multiple predictive approaches, these consensus models also minimised any biases or limitations that could be inherent in single-algorithm models. The exercise showed that consensus modelling, especially when involving a diversified set of ML algorithms, can serve as a powerful tool for enhancing the reliability and accuracy of predictions in the complex field of nanotechnology.

## Supporting Information

File 1 The dataset used to develop the five individual models. The NMs used in training and test sets are also indicated.

File 2 Details of the *k*NN/read-across model presented following the QMRF format.

File 3 Details of the random forest model presented following the QMRF format.

File 4 Details of the AdaBoost regression model presented following the QMRF format.

File 5 Details of the stacked PLS and MLP q-RASPR models presented following the QMRF format.

## Data Availability

All data are available in the Supporting Information.
