# Supplementary material for: The round-robin approach applied to nanoinformatics: consensus prediction of nanomaterials zeta potential
Source: Beilstein J Nanotechnol. 2024 Nov 29;15:1536–53. doi: 10.3762/bjnano.15.121 (PMC11610486; doi:10.3762/bjnano.15.121)
Supplement: File 2 — Details of the kNN/read-across model presented following the QMRF format. [file Beilstein_J_Nanotechnol-15-1536-s002.pdf]

BEILSTEIN JOURNAL OF NANOTECHNOLOGY

Supporting Information

for

The round-robin approach applied to nanoinformatics: consensus prediction of nanomaterials zeta potential

Dimitra-Danai Varsou, Arkaprava Banerjee, Joyita Roy, Kunal Roy, Giannis Savvas, Haralambos Sarimveis, Ewelina Wyrzykowska, Mateusz Balicki, Tomasz Puzyn, Georgia Melagraki, Iseult Lynch and Antreas Afantitis

Beilstein J. Nanotechnol. **2024,** *15,* 1536–1553. doi:10.3762/bjnano.15.121

Details of the *k*NN/read-across model presented following the QMRF format

License and Terms: This is a supporting information file under the terms of the Creative Commons Attribution License (https://creativecommons.org/licenses/by/4.0). Please note that the reuse, redistribution and reproduction in particular requires that the author(s) and source are credited and that individual graphics may be subject to special legal provisions.
The license is subject to the *Beilstein Journal of Nanotechnology* terms and conditions: (https://www.beilstein-journals.org/bjnano/terms)

Annex I –(Q)SAR model reporting format (QMRF) v.2.1

This is an adaptation of an original work by the OECD. The opinions expressed and arguments employed in this adaptation are the sole responsibility of the author(s) of the adaptation and should not be reported as representing the official views of the OECD or of its Member countries. The original source file is https://www.oecd.org/chemicalsafety/risk-assessment/qsar-assessment-framework-annex-1-qsar-model-reporting-format.docx. This content is not subject to CC BY 4.0.

	Element	Explanation
**1.**	**QSAR identifier**	
1.1.	QSAR identifier (title)	Read-across model for the prediction of zeta-potential of metal and metal-oxide nanomaterials (NMs) in aqueous environments.
1.2	Other related models	MS³bD Zeta Potential Predictive Model (https://mszeta.cloud.nanosolveit.eu/)
1.3.	Software coding the model	Isalos Analytics Platform (https://isalos.novamechanics.com/)
**2.**	**General information**	
2.0	Abstract	A kNN/read-across model for the prediction of zeta-potential inorganic NMs in water.
2.1.	Date of QMRF	28 February 2024
2.2.	QMRF author(s) and contact details	NovaMechanics Ltd. (info@novamechanics.com)
2.3.	Date of QMRF update(s)	Not applicable
2.4.	QMRF update(s)	Not applicable
2.5.	Model developer(s) and contact details	Dimitra-Danai Varsou (varsou@novamechanics.com)<br>Antreas Afantitis (afantitis@novamechanics.com)
2.6.	Date of model development and/or publication	2024
2.7.	Reference(s) to main scientific papers and/or software package	Varsou, D.-D.; Banerjee, A.; Roy, J.; Roy, K.; Savvas, G.; Sarimveis, H.; Wyrzykowska, E.; Balicki, M.; Puzyn, T.; Melagraki, G.; Lynch, I.; Afantitis, A. Beilstein Arch. 2024, 202433. doi:10.3762/bxiv.2024.33.v1
2.8.	Availability of information about the model	The model is proprietary: the source code is not available; however, the description of the modelling workflow is presented in the original research article and training and test sets are available as supplementary information of the original research article.

2.9.	Availability of another QMRF for exactly the same model	Not applicable
**3**	**Defining the endpoint - OECD Principle 1: "A DEFINED ENDPOINT"**	**PRINCIPLE 1: "A DEFINED ENDPOINT". ENDPOINT refers to any physicochemical, biological, or environmental property/activity/effect that can be measured and therefore modelled. The intent of PRINCIPLE 1 (a (Q)SAR should be associated with a defined endpoint) is to ensure clarity in the endpoint being predicted by a given model, since a given endpoint could be determined by different experimental protocols and under different experimental conditions. It is therefore important to identify the experimental system and test conditions that is being modelled by the (Q)SAR.**
3.1.	Species	Not applicable
3.2.	Endpoint	Zeta potential in aqueous environment (pH=6.5-8.5).
3.3	Comment on endpoint	Not applicable
3.4.	Endpoint units	Millivolts (mV)
3.5.	Dependent variable	Not applicable
3.6.	Experimental protocol	NanoMILE protocol.
3.7.	Endpoint data quality and variability	The NM physicochemical characterisation data were all generated within the Framework Program 7 (FP7) project NanoMILE, which minimizes the risk of protocol variation.
**4**	**Defining the algorithm - OECD Principle 2 : "AN UNAMBIGUOUS ALGORITHM"**	**PRINCIPLE 2: "AN UNAMBIGUOUS ALGORITHM". The (Q)SAR estimate of an endpoint is the result of applying an ALGORITHM to a set of structural parameters which describe the chemical structure. The intent of PRINCIPLE 2 (a (Q)SAR should be associated with an unambiguous algorithm) is to ensure transparency in the model algorithm that generates predictions of an endpoint from information on chemical structure and/or physicochemical properties. In this context, algorithm refers to any mathematical equation, decision rule or output approach.**
4.1.	Type of model	Type of model: Instance based/read-across, *k*-Nearest Neighbours (*k*NN)
4.2.	Explicit algorithm	The *k*NN/read-across model employs the *k*-nearest neighbours approach, an instance-based method that predicts the endpoint of a sample based on its *k* nearest neighbours (*k* = 7) in the data space. The proximity between samples is measured using Euclidean distance, which is adjusted slightly for categorical descriptor values using a binary value (0 in the case of same class data points or 1, otherwise). The endpoint prediction, in this case the zeta potential value, is the weighted average of the endpoint values of the *k* closest neighbours, with each neighbour's weighting factor inversely proportional to its distance from the evaluated sample.
4.3.	Descriptors in the model	• Equivalent sphere diameter [nm],<br>• Shape group,<br>• Coating,<br>• Hydrodynamic diameter (DLS) [nm],<br>• Hamaker constant of the NMs calculated in water [x E-20 J],<br>• Number of oxygen atoms.

4.4.	Descriptor selection	To identify the most relevant parameters, eliminate noise, and avoid overfitting, the *BestFirst* method with the *CfsSubset* evaluator were employed. The initial pool of descriptors is presented in §4.5. Four descriptors were selected to use in the model: the NMs coating, their equivalent sphere diameter, their hydrodynamic diameter, and the number oxygen atoms present in the core's chemical formula. To enhance the model's performance and interpretability, the Hamaker constant of the NMs calculated in water and the shape group were added to the subset of the selected descriptors.
4.5.	Algorithm and descriptor generation	The physicochemical descriptors were derived by the NMs physicochemical characterization performed under the EU-FP7 NanoMILE project and from the available descriptors/properties, four were included in this study due to completeness of the data (absence of data gaps) regarding: the NMs core chemistry, coating, morphology and hydrodynamic size measured using dynamic light scattering (DLS). To enrich the library of the NMs physicochemical properties and increase the amount of available information, the corresponding sphere diameter (the diameter of the sphere with surface area equal to the area of the NM) was calculated, as well as three molecular descriptors commonly used in nanoinformatics studies. These descriptors were chemical formula-related descriptors (the number of metal and oxygen atoms present in the core's chemical formula, and the molecular weight of the core's compound). Finally, the Hamaker constants of the ENMs were calculated in vacuum and in water using the NanoSolveIT Hamaker tool. These calculations, performed considering spherical and uncoated ENMs, aimed to quantify the attractive (positive values) or repulsive (negative values) interactions between ENMs, leading to agglomeration or aggregation phenomena. In fact, the balance between the Hamaker constant (expressing van der Waals attraction between particles) and the zeta potential values of particles (expressing their electrostatic repulsion) controls the stability of colloidal dispersions according to the DLVO theory. For the computational analysis, the TIP3P force field was employed for water, while the Dreiding force field was utilized for the ENMs. In the case of Zr-doped CeO_2 ENMs ($Ce_xZr_yO_2$), the same density as for pure CeO_2 ENMs was considered to maintain consistency.
4.6.	Software name and version for descriptor generation	The Hammaker constants were calculated in vacuum and in water using the NanoSolveIT Hamaker tool (https://hamaker.cloud.nanosolveit.eu/).
4.7.	Chemicals/Descriptors ratio	53 training NMs/ 6 descriptors.
**5**	**Defining the applicability domain - OECD Principle 3: "A DEFINED DOMAIN OF APPLICABILITY"**	**PRINCIPLE 3: "A DEFINED DOMAIN OF APPLICABILITY". APPLICABILITY DOMAIN refers to the response and chemical structure space in which the model makes predictions with a given reliability. Ideally the applicability domain should express the structural, physicochemical and response space of the model. The CHEMICAL STRUCTURE (x variable) space can be expressed by information on physicochemical properties and/or structural fragments. The RESPONSE (y variable) can be any physicochemical, biological or environmental effect that is being predicted. According to PRINCIPLE 3 a (Q)SAR should be associated with a defined domain of applicability. Section 5 can be repeated (e.g., 5.a, 5.b, 5.c, etc) as many times as necessary if more than one method has been used to assess the applicability domain.**

5.1.	Description of the applicability domain of the model	The applicability domain was defined based on the leverage method. The necessary calculations were performed based on the numerical descriptors of the train set.
5.2.	Method used to assess the applicability domain	In the leverage method, the leverage values h -which are the diagonal elements of the Hat matrix- reflect the similarity of the validation or untested samples to the training set (distance from the training set's centroid) based on the descriptor values used in the model development. The limits of the AD are determined by the threshold leverage value h*. The prediction for a validation or untested NP is considered reliable if h<h*.
5.3.	Software name and version for applicability domain assessment	Isalos Analytics Platform, domain-leverage functionality
5.4.	Limits of applicability	The leverage threshold was calculated based on the training NMs subset and set to 0.226.
**6**	**Defining goodness-of-fit and robustness (internal validation) – OECD Principle 4: "APPROPRIATE MEASURES OF GOODNESS-OF-FIT, ROBUSTENESS AND PREDICTIVITY"**	**PRINCIPLE 4: "APPROPRIATE MEASURES OF GOODNESS-OF-FIT, ROBUSTENESS AND PREDICTIVITY". PRINCIPLE 4 expresses the need to perform validation to establish the performance of the model. GOODNESS-OF-FIT and ROBUSTNESS refer to the internal model performance.**
6.1.	Availability of the training set	The training set is available as a supporting information file of the relevant publication.
6.2.	Available information for the training set	NMs dataset including physicochemical characterisation, molecular descriptors and Hamaker constants.
6.3.	Data for each descriptor variable for the training set	The training set is available as a supporting information file of the relevant publication.
6.4.	Data for the dependent variable for the training set	The training set is available as a supporting information file of the relevant publication.
6.5.	Other information about the training set	The training set comprises of 53 NMs randomly selected from the pool of the original NMs.
6.6.	Pre-processing of data before modelling	Z-score (Gaussian) normalization of the independent variables
6.7.	Statistics for goodness-of-fit	R^2 = 0.99<br>Adjusted R^2 = 0.99<br>MAE = 0.29<br>RMSE = 0.54
6.8.	Robustness - Statistics obtained by leave-one-out cross-validation	Robustness – Statistics obtained by leave-one-out cross-validation (training set):<br>R^2 = 0.62<br>Adjusted R^2 = 0.62<br>MAE = 12.09<br>RMSE = 20.05

6.9.	Robustness - Statistics obtained by leave-many-out cross-validation	Robustness – Statistics obtained by five-fold cross-validation (random selection, training set):<br>R^2 = 0.66<br>Adjusted R^2 = 0.66<br>MAE = 13.12<br>RMSE = 19.13
6.10.	Robustness - Statistics obtained by Y-scrambling	Robustness – Statistics obtained by Y-scrambling:<br>**#** **R^2** **RMSE**<br>1 -0.74 36.40<br>2 0.28 23.45<br>3 -0.51 33.97<br>4 0.30 23.06<br>5 -0.34 32.00<br>6 -1.47 43.39<br>7 -0.33 31.85<br>8 -1.33 42.20<br>9 -0.33 31.83<br>10 -0.35 32.05
6.11.	Robustness - Statistics obtained by bootstrap	Not applicable
6.12.	Robustness - Statistics obtained by other methods	Not applicable
**7**	**Defining predictivity (external validation) – OECD Principle 4: “APPROPRIATE MEASURES OF GOODNESS-OF-FIT, ROBUSTENESS AND PREDICTIVITY”**	**PRINCIPLE 4: “APPROPRIATE MEASURES OF GOODNESS-OF-FIT, ROBUSTENESS AND PREDICTIVITY”. PRINCIPLE 4 expresses the need to perform validation to establish the performance of the model. PREDICTIVITY refers to the external model validation. Section 7 can be repeated (e.g., 7.a, 7.b, 7.c, etc) as many times as necessary if more validation studies need to be reported in the QMRF.**
7.1.	Availability of the external validation set	The test set is available as a supporting information file of the relevant publication.
7.2.	Available information for the external validation set	Nanomaterials dataset.
7.3.	Data for each descriptor variable for the external validation set	The test set is available as a supporting information file of the relevant publication.
7.4.	Data for the dependent variable for the external validation set	The test set is available as a supporting information file of the relevant publication.
7.5.	Other information about the external validation set	External test set with 18 compounds appended.
7.6.	Experimental design of test set	Random selection of NM samples before modelling (25% of the original set).

<table>
<tr><td>7.7.</td><td>Predictivity - Statistics obtained by external validation</td><td>R^2 = 0.88
Adjusted R^2 = 0.88
MAE = 7.81
RMSE = 9.71
Q^2_{ext} = 0.88

Golbraikh and Tropsha[1] test results:
<table>
<tr><th>Criterion</th><th>Assessment</th><th>Result</th></tr>
<tr><td>R^2 > 0.6</td><td>PASS</td><td>R^2 = 0.894</td></tr>
<tr><td>Rcvext^2 > 0.5</td><td>PASS</td><td>Rcvext^2 = 0.884</td></tr>
<tr><td>(R^2 - R0^2)/R^2 < 0.1</td><td>PASS</td><td>(R^2 - R0^2)/R^2 = 0.001</td></tr>
<tr><td>(R^2 - R'0^2)/R^2 < 0.1</td><td>PASS</td><td>(R^2 - R'0^2)/R^2 = 0.002</td></tr>
<tr><td>abs(R0^2 - R'0^2) < 0.3</td><td>PASS</td><td>abs(R0^2 - R'0^2) = 0.001</td></tr>
<tr><td>0.85 < k < 1.15</td><td>PASS</td><td>k = 0.883</td></tr>
<tr><td>0.85 < k' < 1.15</td><td>PASS</td><td>k' = 1.012</td></tr>
<tr><td colspan="3">Model Predictive</td></tr>
</table>
</td></tr>
<tr><td>7.8.</td><td>Predictivity - Assessment of the external validation set</td><td>The test set is the 25% of the original data. The leverage threshold was calculated based on the training NMs subset and set to 0.226. The test NM samples had values within the range of 0.031 to 0.191, indicating that their predictions were reliable except the one NM sample whose leverage value was equal to 0.859.</td></tr>
<tr><td>7.9.</td><td>Comments on the external validation of the model</td><td>Not applicable</td></tr>
<tr><td>8</td><td>Providing a mechanistic interpretation - OECD Principle 5: “A MECHANISTIC INTERPRETATION, IF POSSIBLE”</td><td>PRINCIPLE 5: “A MECHANISTIC INTERPRETATION, IF POSSIBLE”. According to PRINCIPLE 5, a (Q)SAR should be associated with a mechanistic interpretation, if possible.</td></tr>
<tr><td>8.1.</td><td>Mechanistic basis of the model</td><td>The Stern and diffusion layers, as well as the distance from the bare NM's surface where its charge will persist, are determined by the NM's core size. The NM coating influences the measured zeta potential. If the coating is sufficiently thick, it may disguise the bare NM's surface charge, and the observed zeta potential will be determined by the coating charge. If the coating is not thick enough, the observed zeta potential will be caused by an interaction between the coating charge and thickness and the base NM surface charge. The shape is also a critical parameter for NMs zeta potential: non-spherical particles can exhibit slightly different zeta potential values compared to spherical ones due to varying surface area distribution and potential interactions between different facets of the NM. The balance between the Hamaker constant (expressing van der Waals attraction between particles) and the ZP values of particles (expressing their electrostatic repulsion) controls the stability of colloidal dispersions according to the DLVO theory.</td></tr>
<tr><td>8.2.</td><td>A priori or a posteriori mechanistic interpretation</td><td>A posteriori mechanistic interpretation.</td></tr>
</table>

[1] i) Golbraikh, A. & Tropsha, A. Beware of q^2! *J. Mol. Graph. Model.* **20**, 269–276 (2002). ii) Tropsha, A., Gramatica, P. & Gombar, V. K. The importance of being earnest: Validation is the absolute essential for successful application and interpretation of QSPR models. *Qsar Comb. Sci.* **22**, 69–77 (2003). iii) Melagraki, G. & Afantitis, A. Enalos KNIME nodes: Exploring corrosion inhibition of steel in acidic medium. *Chemom. Intell. Lab. Syst.* **123**, 9–14 (2013).

8.3.	Other information about the mechanistic interpretation	Not applicable
**9**	**Miscellaneous information**	
9.1.	Comments	Not applicable
9.2.	Bibliography	Not applicable
9.3	Supporting information	Not applicable
