# Supplementary material for: The round-robin approach applied to nanoinformatics: consensus prediction of nanomaterials zeta potential
Source: Beilstein J Nanotechnol. 2024 Nov 29;15:1536–53. doi: 10.3762/bjnano.15.121 (PMC11610486; doi:10.3762/bjnano.15.121)
Supplement: File 3 — Details of the random forest model presented following the QMRF format. [file Beilstein_J_Nanotechnol-15-1536-s003.pdf]

BEILSTEIN JOURNAL OF NANOTECHNOLOGY

Supporting Information

for

The round-robin approach applied to nanoinformatics: consensus prediction of nanomaterials zeta potential

Dimitra-Danai Varsou, Arkaprava Banerjee, Joyita Roy, Kunal Roy, Giannis Savvas, Haralambos Sarimveis, Ewelina Wyrzykowska, Mateusz Balicki, Tomasz Puzyn, Georgia Melagraki, Iseult Lynch and Antreas Afantitis

Beilstein J. Nanotechnol. **2024,** *15,* 1536–1553. doi:10.3762/bjnano.15.121

Details of the random forest model presented following the QMRF format

License and Terms: This is a supporting information file under the terms of the Creative Commons Attribution License (https://creativecommons.org/licenses/by/4.0). Please note that the reuse, redistribution and reproduction in particular requires that the author(s) and source are credited and that individual graphics may be subject to special legal provisions.
The license is subject to the *Beilstein Journal of Nanotechnology* terms and conditions: (https://www.beilstein-journals.org/bjnano/terms)

Annex I –(Q)SAR model reporting format (QMRF) v.2.1

This is an adaptation of an original work by the OECD. The opinions expressed and arguments employed in this adaptation are the sole responsibility of the author(s) of the adaptation and should not be reported as representing the official views of the OECD or of its Member countries. The original source file is https://www.oecd.org/chemicalsafety/risk-assessment/qsar-assessment-framework-annex-1-qsar-model-reporting-format.docx. This content is not subject to CC BY 4.0.

QMRF v.2.1 is a minor update of the QMRF template, as it only concerns the description of the QMRF fields. The only exception is Section 10, which has been entirely removed. This section referred to the JRC QSAR Model Database, which is not updated anymore.

The update is based on the version 2.0[1].

	Element	Explanation
**1.**	**QSAR identifier**	
1.1.	QSAR identifier (title)	Random Forest Regressor model for zeta potential of engineered nanomaterials
1.2	Other related models	-NA-
1.3.	Software coding the model	Python, Google Colab using scikit-learn, pandas, NumPy libraries.

[1] Triebe, J., Worth, A., Janusch Roi, A. and Coe, A., JRC QSAR Model Database: EURL ECVAM DataBase service on ALternative Methods to animal experimentation: To promote the development and uptake of alternative and advanced methods in toxicology and biomedical sciences: User Support & Tutorial, EUR 28713 EN, Publications Office of the European Union, Luxembourg, 2017, ISBN 978-92-79-71406-1, doi:10.2760/905519, JRC107491.

2.	General information	
2.0	Abstract	A random forest Regressor was developed for predicting the zeta potential of engineered nanomaterials. Feature selection using Pearson corelation as well as recursive feature elimination was conducted to find the optimal set of features. Grid search algorithm was also implemented to find the best model parameters. After finding the best parameters, along with the best features, they were used to make predictions on the test set.
2.1.	Date of QMRF	6th March 2024
2.2.	QMRF author(s) and contact details	Sarimveis Charalampos, Tsiros Periklis, Savvas Ioannis<br>yiannisn2000@gmail.com
2.3.	Date of QMRF update(s)	-NA-
2.4.	QMRF update(s)	-NA-
2.5.	Model developer(s) and contact details	Sarimveis Charalampos, Tsiros Periklis, Savvas Ioannis<br>yiannisn2000@gmail.com
2.6.	Date of model development and/or publication	2024
2.7.	Reference(s) to main scientific papers and/or software package	[1] Roy K, Mitra I. On various metrics used for validation of predictive QSAR models with applications in virtual screening and focused library design. Comb Chem High Throughput Screen. 2011 Jul;14(6):450-74. doi: 10.2174/138620711795767893. PMID: 21521150.<br><br>[2] Tropsha, A., Gramatica, P. and Gombar, V. K., The Importance of Being Earnest: Validation is the Absolute Essential for Successful Application and Interpretation of QSPR Models. Quantitative Structure Activity Relationships, 22 (2003) 1–9.<br><br>[3] Golbraikh A, Tropsha A. Beware of q2! J Mol Graph Model. 2002 Jan;20(4):269-76. doi: 10.1016/s1093-3263(01)00123-1. PMID: 11858635.
2.8.	Availability of information about the model	The model is non-proprietary: Full description of the model algorithm is available, training and test sets are available as supplementary material of original research article.
2.9.	Availability of another QMRF for exactly the same model	NO

**3**	**Defining the endpoint - OECD Principle 1: "A DEFINED ENDPOINT"**	**PRINCIPLE 1: "A DEFINED ENDPOINT". ENDPOINT refers to any physicochemical, biological, or environmental property/activity/effect that can be measured and therefore modelled. The intent of PRINCIPLE 1 (a (Q)SAR should be associated with a defined endpoint) is to ensure clarity in the endpoint being predicted by a given model, since a given endpoint could be determined by different experimental protocols and under different experimental conditions. It is therefore important to identify the experimental system and test conditions that is being modelled by the Q)SAR.**
3.1.	Species	-NA-
3.2.	Endpoint	Zeta potential of engineered nanomaterials.
3.3	Comment on endpoint	-NA-
3.4.	Endpoint units	-mV-
3.5.	Dependent variable	Zeta potential of engineered nanomaterials. Scaling of the dependant variable was not performed.
3.6.	Experimental protocol	-NA-
3.7.	Endpoint data quality and variability	The physicochemical data were obtained under the EU-FP7 NanoMILE project. The Zeta Potential was measured in water (pH=6.5-8.5).

**4**	**Defining the algorithm - OECD Principle 2 : "AN UNAMBIGUOUS ALGORITHM"**	**PRINCIPLE 2: "AN UNAMBIGUOUS ALGORITHM". The (Q)SAR estimate of an endpoint is the result of applying an ALGORITHM to a set of structural parameters which describe the chemical structure. The intent of PRINCIPLE 2 (a (Q)SAR should be associated with an unambiguous algorithm) is to ensure transparency in the model algorithm that generates predictions of an endpoint from information on chemical structure and/or physicochemical properties. In this context, algorithm refers to any mathematical equation, decision rule or output approach.**
4.1.	Type of model	Machine Learning Model: (Random Forest Regressor)
4.2.	Explicit algorithm	Random Forest Regressor was chosen to be the model algorithm. Feature selection and recursive feature elimination was conducted to find optimal set of features. Categorical Descriptors were one-hot-encoded so that they were able to be processed by the algorithm. Also, hyperparameter tuning was implemented using grid-search algorithm. After finding the best model parameters and features they were used to make predictions on the test set.
4.3.	Descriptors in the model	Equivalent sphere diameter [nm], Coating (One hot encoded), DLS (hydrodymic diam.) [nm], MW [g/mol]
4.4.	Descriptor selection	Initial number of descriptors were 11. Three of them were preproccessed for one-hot-encoding. Pearson correlation was calculated for each pair of features. One feature was dropped because of very high correlation with another one (0.97). Recursive feature elimination was then performed, and non-important descriptors were dropped. Final number of descriptors selected for modelling were 8. Coating descriptor is 5 collumns because of one hot encoding.
4.5.	Algorithm and descriptor generation	-NA-
4.6.	Software name and version for descriptor generation	-NA-
4.7.	Chemicals/Descriptors ratio	53/8

**5**	**Defining the applicability domain - OECD Principle 3: “A DEFINED DOMAIN OF APPLICABILITY”**	**PRINCIPLE 3: “A DEFINED DOMAIN OF APPLICABILITY”. APPLICABILITY DOMAIN refers to the response and chemical structure space in which the model makes predictions with a given reliability. Ideally the applicability domain should express the structural, physicochemical and response space of the model. The CHEMICAL STRUCTURE (x variable) space can be expressed by information on physicochemical properties and/or structural fragments. The RESPONSE (y variable) can be any physicochemical, biological or environmental effect that is being predicted. According to PRINCIPLE 3 a (Q)SAR should be associated with a defined domain of applicability. Section 5 can be repeated (e.g., 5.a, 5.b, 5.c, etc) as many times as necessary if more than one method has been used to assess the applicability domain.**
5.1.	Description of the applicability domain of the model	The applicability domain was defined using the Leverage method to identify outliers.
5.2.	Method used to assess the applicability domain	Leverage approach
5.3.	Software name and version for applicability domain assessment	Jaqpotpy
5.4.	Limits of applicability	Leverage threshold is $h^* = 0.509$. Compounds with a value above the threshold are considered outside of the applicability domain. In the training set one compound had $h = 0.54 > h^*$. In the test set, one compound had $h = 0.94$. The predictions of those 2 points thus are not considered reliable.

**6**	**Defining goodness-of-fit and robustness (internal validation) – OECD Principle 4: “APPROPRIATE MEASURES OF GOODNESS-OF-FIT, ROBUSTENESS AND PREDICTIVITY”**	**PRINCIPLE 4: “APPROPRIATE MEASURES OF GOODNESS-OF-FIT, ROBUSTENESS AND PREDICTIVITY”. PRINCIPLE 4 expresses the need to perform validation to establish the performance of the model. GOODNESS-OF-FIT and ROBUSTNESS refer to the internal model performance.**
6.1.	Availability of the training set	Available in the Supplementary Information
6.2.	Available information for the training set	Available information for the training set: Descriptors for the nanomaterials.
6.3.	Data for each descriptor variable for the training set	Available in the Supplementary Information
6.4.	Data for the dependent variable for the training set	Available in the Supplementary Information
6.5.	Other information about the training set	-NA-
6.6.	Pre-processing of data before modelling	One hot encoding categorical (string) values. No data scaling need because of Random Forest Regressor algorithm.
6.7.	Statistics for goodness-of-fit	-NA-
6.8.	Robustness - Statistics obtained by leave-one-out cross-validation	Q2(LOO) = 0.611
6.9.	Robustness - Statistics obtained by leave-many-out cross-validation	-NA-
6.10.	Robustness - Statistics obtained by Y-scrambling	-NA-

6.11.	Robustness - Statistics obtained by bootstrap	-NA-
6.12.	Robustness - Statistics obtained by other methods	-NA-
**7**	**Defining predictivity (external validation) – OECD Principle 4: “APPROPRIATE MEASURES OF GOODNESS-OF-FIT, ROBUSTENESS AND PREDICTIVITY”**	**PRINCIPLE 4: “APPROPRIATE MEASURES OF GOODNESS-OF-FIT, ROBUSTENESS AND PREDICTIVITY”. PRINCIPLE 4 expresses the need to perform validation to establish the performance of the model. PREDICTIVITY refers to the external model validation. Section 7 can be repeated (e.g., 7.a, 7.b, 7.c, etc) as many times as necessary if more validation studies need to be reported in the QMRF.**
7.1.	Availability of the external validation set	Available
7.2.	Available information for the external validation set	Available information for the test set: Descriptors for the nanomaterials.
7.3.	Data for each descriptor variable for the external validation set	Available as supporting information
7.4.	Data for the dependent variable for the external validation set	Available as supporting information
7.5.	Other information about the external validation set	18 total instances of engineered nanomaterials
7.6.	Experimental design of test set	-NA-
7.7.	Predictivity - Statistics obtained by external validation	R^2(coefficient of determination) = 0.941<br>R^2_{cv}(external) = 0.944<br>MAE = 5.426<br>RMSE = 6.733<br>Golbraiky and Tropsha Criteria<br><br>R^2 > 0.6 (correlation coeff) | PASS | R^2 = 0.941<br>Q^2(LOO) > 0.5 | PASS | Q^2(LOO) = 0.611<br>(R^2 - R0^2)/R^2 <0.1 | PASS | (R^2 - R0^2)/R^2 = - 0.063<br>(R^2 - R'0^2)/R^2 <0.1 | PASS | (R^2 - R'0^2)/R^2 = - 0.058<br>abs(R0^2-R'0^2) < 0.3 | PASS | abs(R0^2-R'0^2) = 0.004<br>0.85 < k < 1.15 | PASS | k = 1.006<br>0.85 < k' <1.15 | PASS | k’ = 0.936

7.8.	Predictivity - Assessment of the external validation set	The external validation set is large, containing 18 nanomaterials. It is representative of the applicability domain. 17 nanomaterials lie inside the AD while one is above the DOA threshold.
7.9.	Comments on the external validation of the model	Predictive
**8**	**Providing a mechanistic interpretation - OECD Principle 5: “A MECHANISTIC INTERPRETATION, IF POSSIBLE”**	**PRINCIPLE 5: “A MECHANISTIC INTERPRETATION, IF POSSIBLE”. According to PRINCIPLE 5, a (Q)SAR should be associated with a mechanistic interpretation, if possible.**
8.1.	Mechanistic basis of the model	-NA-
8.2.	A priori or a posteriori mechanistic interpretation	-NA-
8.3.	Other information about the mechanistic interpretation	-NA-
**9**	**Miscellaneous information**	
9.1.	Comments	None
9.2.	Bibliography	1] Roy K, Mitra I. On various metrics used for validation of predictive QSAR models with applications in virtual screening and focused library design. Comb Chem High Throughput Screen. 2011 Jul;14(6):450-74. doi: 10.2174/138620711795767893. PMID: 21521150.

		[2] Tropsha, A., Gramatica, P. and Gombar, V. K., The Importance of Being Earnest: Validation is the Absolute Essential for Successful Application and Interpretation of QSPR Models. Quantitative Structure Activity Relationships, 22 (2003) 1–9.<br><br>[3] Golbraikh A, Tropsha A. Beware of q2! J Mol Graph Model. 2002 Jan;20(4):269-76. doi: 10.1016/s1093-3263(01)00123-1. PMID: 11858635.
9.3	Supporting information	Training and test datasets are attached as Supplementary Information.
