# Supplementary material for: The round-robin approach applied to nanoinformatics: consensus prediction of nanomaterials zeta potential
Source: Beilstein J Nanotechnol. 2024 Nov 29;15:1536–53. doi: 10.3762/bjnano.15.121 (PMC11610486; doi:10.3762/bjnano.15.121)
Supplement: File 5 — Details of the stacked PLS and MLP q-RASPR models presented following the QMRF format. [file Beilstein_J_Nanotechnol-15-1536-s005.pdf]

BEILSTEIN JOURNAL OF NANOTECHNOLOGY

Supporting Information

for

The round-robin approach applied to nanoinformatics: consensus prediction of nanomaterials zeta potential

Dimitra-Danai Varsou, Arkaprava Banerjee, Joyita Roy, Kunal Roy, Giannis Savvas, Haralambos Sarimveis, Ewelina Wyrzykowska, Mateusz Balicki, Tomasz Puzyn, Georgia Melagraki, Iseult Lynch and Antreas Afantitis

Beilstein J. Nanotechnol. **2024,** *15,* 1536–1553. doi:10.3762/bjnano.15.121

Details of the stacked PLS and MLP q-RASPR models presented following the QMRF format

License and Terms: This is a supporting information file under the terms of the Creative Commons Attribution License (https://creativecommons.org/licenses/by/4.0). Please note that the reuse, redistribution and reproduction in particular requires that the author(s) and source are credited and that individual graphics may be subject to special legal provisions.
The license is subject to the *Beilstein Journal of Nanotechnology* terms and conditions: (https://www.beilstein-journals.org/bjnano/terms)

Annex I –(Q)SAR model reporting format (QMRF) v.2.1

This is an adaptation of an original work by the OECD. The opinions expressed and arguments employed in this adaptation are the sole responsibility of the author(s) of the adaptation and should not be reported as representing the official views of the OECD or of its Member countries. The original source file is https://www.oecd.org/chemicalsafety/risk-assessment/qsar-assessment-framework-annex-1-qsar-model-reporting-format.docx. This content is not subject to CC BY 4.0.

QMRF v.2.1 is a minor update of the QMRF template, as it only concerns the description of the QMRF fields. The only exception is Section 10, which has been entirely removed. This section referred to the JRC QSAR Model Database, which is not updated anymore.

The update is based on the version 2.0[1].

	Element	Explanation
**1.**	**QSAR identifier**	
1.1.	QSAR identifier (title)	1. Stacked PLS q-RASPR model for zeta potential of engineered nanomaterials<br>2. Stacked MLP q-RASPR model for zeta potential of engineered nanomaterials
1.2	Other related models	MLR q-RASPR models (M1-M4) for zeta potential of engineered nanomaterials
1.3.	Software coding the model	PLS_SingleY_1.0 (Roy's group), Jupyter Notebook using Scikit-learn

[1] Triebe, J., Worth, A., Janusch Roi, A. and Coe, A., JRC QSAR Model Database: EURL ECVAM DataBase service on ALternative Methods to animal experimentation: To promote the development and uptake of alternative and advanced methods in toxicology and biomedical sciences: User Support & Tutorial, EUR 28713 EN, Publications Office of the European Union, Luxembourg, 2017, ISBN 978-92-79-71406-1, doi:10.2760/905519, JRC107491.

2.	General information	
2.0	Abstract	Four MLR q-RASPR models for zeta potential of engineered nanomaterials were developed after suitable feature selection. The predictions for these models were taken as descriptors to develop Stacked PLS and MLP q-RASPR models for zeta potential of engineered nanomaterials
2.1.	Date of QMRF	29th February 2024
2.2.	QMRF author(s) and contact details	Arkaprava Banerjee, Joyita Roy, Kunal Roy*<br>kunal.roy@jadavpuruniversity.in
2.3.	Date of QMRF update(s)	-NA-
2.4.	QMRF update(s)	-NA-
2.5.	Model developer(s) and contact details	Arkaprava Banerjee, Joyita Roy<br>kunal.roy@jadavpuruniversity.in
2.6.	Date of model development and/or publication	2024
2.7.	Reference(s) to main scientific papers and/or software package	1. Banerjee A, Roy K. Mol Divers 2022, 26, 2847-2862<br>2. Banerjee A, Gajewicz-Skretna A, Roy K. Mol Inform 2023, 42, 2200261<br>3. Banerjee A, Kar S, Pore S, Roy K. Nanotoxicology 2023, 17, 78-93<br>4. Wold S, Sjostrom M, Eriksson L. Chemom Intell Lab Syst 2001, 58, 109-130<br>5. Hackeling G. Mastering Machine Learning with scikit-learn. Packt Publishing Ltd; 2017
2.8.	Availability of information about the model	The model is non-proprietary: A full description of the model algorithm is available, and training and test sets are available as the Supplementary material of the original research article
2.9.	Availability of another QMRF for exactly the same model	No

**3**	**Defining the endpoint - OECD Principle 1: “A DEFINED ENDPOINT"**	**PRINCIPLE 1: “A DEFINED ENDPOINT". ENDPOINT refers to any physicochemical, biological, or environmental property/activity/effect that can be measured and therefore modelled. The intent of PRINCIPLE 1 (a (Q)SAR should be associated with a defined endpoint) is to ensure clarity in the endpoint being predicted by a given model, since a given endpoint could be determined by different experimental protocols and under different experimental conditions. It is therefore important to identify the experimental system and test conditions that is being modelled by the Q)SAR.**
3.1.	Species	-NA-
3.2.	Endpoint	Zeta potential of engineered nanomaterials
3.3	Comment on endpoint	-NA-
3.4.	Endpoint units	mV
3.5.	Dependent variable	Zeta potential of engineered nanomaterials was the dependent variable that was modelled. No logarithmic transformation was done.
3.6.	Experimental protocol	-NA-.
3.7.	Endpoint data quality and variability	The physicochemical data were obtained under the EU-FP7 NanoMILE project. The Zeta Potential was measured in water (pH=6.5-8.5).

**4**	**Defining the algorithm - OECD Principle 2 : "AN UNAMBIGUOUS ALGORITHM"**	**PRINCIPLE 2: "AN UNAMBIGUOUS ALGORITHM". The (Q)SAR estimate of an endpoint is the result of applying an ALGORITHM to a set of structural parameters which describe the chemical structure. The intent of PRINCIPLE 2 (a (Q)SAR should be associated with an unambiguous algorithm) is to ensure transparency in the model algorithm that generates predictions of an endpoint from information on chemical structure and/or physicochemical properties. In this context, algorithm refers to any mathematical equation, decision rule or output approach.**
4.1.	Type of model	Equation-based (PLS q-RASPR), Machine Learning model (MLP q-RASPR)
4.2.	Explicit algorithm	After suitable feature selection (QSPR descriptors) from the training set, these features were used to compute the RASPR descriptors. Data fusion was performed, by clubbing the initially selected QSPR descriptors with the computed RASPR descriptors, and a further feature selection algorithm was applied. Four different MLR q-RASPR models were developed using different combinations of selected descriptors. The predictions from these models were used as descriptors to generate PLS and MLP q-RASPR models that were used as the final stacking regressors after optimization of the associated hyperparameters. The detailed algorithm of q-RASAR/q-RASPR can be explored from the following literature sources:<br>1. Banerjee A, Roy K. Mol Divers 2022, 26, 2847-2862<br>2. Banerjee A, Gajewicz-Skretna A, Roy K. Mol Inform 2023, 42, 2200261<br>3. Banerjee A, Roy K. Chem Res Toxicol 2023, 36, 446-464.
4.3.	Descriptors in the model	4 descriptors (Ypred(M1), Ypred(M2), Ypred(M3), Ypred(M4))
4.4.	Descriptor selection	A total of 72 structural, physicochemical, and periodic table QSPR descriptors were initially screened by employing the Stepwise Selection and the Genetic Algorithm approaches. From these 72 descriptors, the Best Subset Selection was applied to select a pool of 10 QSPR descriptors. Additionally we have taken the log-transformed hydrodynamic diameter since we found that it possessed significant correlation with the training set response values. The RASPR descriptors were computed on these (10+1) QSPR descriptors. The generated 18 different RASPR descriptors were clubbed with the selected 11 QSPR descriptors to obtain a pool of 29 descriptors. Feature selection in the form of Best Subset Selection was applied and four different MLR q-RASPR models were generated. The predictions for these four models were then used as descriptors and the final stacked PLS q-RASPR and MLP q-RASPR models were generated.
4.5.	Algorithm and descriptor generation	Structural, physicochemical, and periodic table descriptors (QSPR)<br>Similarity and error-based descriptors (q-RASPR)
4.6.	Software name and version for descriptor generation	Elemental Descriptor Calculator, RASAR-Desc-Calc-v3.0.2

4.7.	Chemicals/Descriptors ratio	53/4
**5**	**Defining the applicability domain - OECD Principle 3: "A DEFINED DOMAIN OF APPLICABILITY"**	**PRINCIPLE 3: "A DEFINED DOMAIN OF APPLICABILITY". APPLICABILITY DOMAIN refers to the response and chemical structure space in which the model makes predictions with a given reliability. Ideally the applicability domain should express the structural, physicochemical and response space of the model. The CHEMICAL STRUCTURE (x variable) space can be expressed by information on physicochemical properties and/or structural fragments. The RESPONSE (y variable) can be any physicochemical, biological or environmental effect that is being predicted. According to PRINCIPLE 3 a (Q)SAR should be associated with a defined domain of applicability. Section 5 can be repeated (e.g., 5.a, 5.b, 5.c, etc) as many times as necessary if more than one method has been used to assess the applicability domain.**
5.1.	Description of the applicability domain of the model	The applicability domain was defined using the Leverage approach that utilizes a HAT matrix and identifies structural outliers. These structural outliers are compounds that are structurally different from the other compounds and thus, do not fall under the chemical space defined by the model. The traditional approach to identifying structural outliers with the leverage approach uses the descriptor values as the source of information, however, since our final regressors are stacked models, we have treated the predicted values of the individual MLR q-RASPR models as descriptors, From the training set, one compound (#28) had a higher leverage value and thus, was considered as an outlier. In the test set, no compounds were outside the AD.
5.2.	Method used to assess the applicability domain	Leverage approach
5.3.	Software name and version for applicability domain assessment	Hi_Calculator-v2.0
5.4.	Limits of applicability	Compounds having leverage values lower than the threshold (h^*=0.283) are considered as inside the applicability domain (AD). From the training set, one compound (#28) had a higher leverage value and thus, was considered as an outlier. In the test set, no compounds were outside the

		AD.
**6**	**Defining goodness-of-fit and robustness (internal validation) – OECD Principle 4: "APPROPRIATE MEASURES OF GOODNESS-OF-FIT, ROBUSTENESS AND PREDICTIVITY"**	**PRINCIPLE 4: "APPROPRIATE MEASURES OF GOODNESS-OF-FIT, ROBUSTENESS AND PREDICTIVITY". PRINCIPLE 4 expresses the need to perform validation to establish the performance of the model. GOODNESS-OF-FIT and ROBUSTNESS refer to the internal model performance.**
6.1.	Availability of the training set	It is available in the Supplementary Information.
6.2.	Available information for the training set	Available information for the training set: Chemical names are available and the data points are for nanomaterials.
6.3.	Data for each descriptor variable for the training set	Available and attached as the Supporting information
6.4.	Data for the dependent variable for the training set	Available and attached as the Supporting information
6.5.	Other information about the training set	The number of training set data points: 53 engineered nanomaterials. The training set was obtained by randomly assigning 75% of the data points from the whole dataset.
6.6.	Pre-processing of data before modelling	Logarithmic transformation of the "hydrodynamic diameter measured by DLS" was performed. The raw data and processed data are not given as per this QMRF; however, the raw data may be available from other QMRFs of this round-robin exercise.
6.7.	Statistics for goodness-of-fit	Stacked PLS q-RASPR: R2=0.681, MAEtrain=13.255, RMSEC=18.417<br>Stacked MLP q-RASPR: R2=0.695, MAEtrain=12.952, RMSEC=18.015
6.8.	Robustness - Statistics obtained by leave-one-out cross-validation	Stacked PLS q-RASPR: Q2(LOO)=0.657. MAE(LOO)=13.766<br>Stacked MLP q-RASPR: Q2(LOO)=0.645, MAE(LOO)=13.957
6.9.	Robustness - Statistics obtained by leave-many-out cross-validation	-NA-

6.10.	Robustness - Statistics obtained by Y-scrambling	-NA-
6.11.	Robustness - Statistics obtained by bootstrap	-NA-
6.12.	Robustness - Statistics obtained by other methods	-NA-
**7**	**Defining predictivity (external validation) – OECD Principle 4: "APPROPRIATE MEASURES OF GOODNESS-OF-FIT, ROBUSTENESS AND PREDICTIVITY"**	**PRINCIPLE 4: "APPROPRIATE MEASURES OF GOODNESS-OF-FIT, ROBUSTENESS AND PREDICTIVITY". PRINCIPLE 4 expresses the need to perform validation to establish the performance of the model. PREDICTIVITY refers to the external model validation. Section 7 can be repeated (e.g., 7.a, 7.b, 7.c, etc) as many times as necessary if more validation studies need to be reported in the QMRF.**
7.1.	Availability of the external validation set	Available
7.2.	Available information for the external validation set	Available information for the test set: Chemical names are available and the data points are for nanomaterials.
7.3.	Data for each descriptor variable for the external validation set	Available and attached as supporting information
7.4.	Data for the dependent variable for the external validation set	Available and attached as supporting information
7.5.	Other information about the external validation set	Number of test set data points: 18 engineered nanomaterials
7.6.	Experimental design of test set	Randomly setting aside chemicals before modeling
7.7.	Predictivity - Statistics obtained by external validation	Stacked PLS q-RASPR: $r_{test}^2 = 0.960, Q_{ext}^2 = 0.951, MAE = 4.402, RMSEP = 6.320$<br>Stacked MLP q-RASPR: $r_{test}^2 = 0.961, Q_{ext}^2 = 0.963, MAE = 4.038, RMSEP = 5.500$

7.8.	Predictivity - Assessment of the external validation set	The external validation set is sufficiently large (n_{test}=18) and representative of the applicability domain (No compounds outside AD (see section 5.1)). The range of descriptors for the training and test sets is as follows:<br><br>Ypred(M1): Training set range=86.657, Test set range=95.196<br>Ypred(M2): Training set range=102.768, Test set range=124.117<br>Ypred(M3): Training set range=98.843, Test set range=111.952<br>Ypred(M4): Training set range=99.156, Test set range=105.526<br><br>The observed response range for the training and test sets is as follows:<br>Yobs: Training set range=114.6 (Max=64.3, Min=-50.3), Test set=98.9 (Max=52.7, Min=-46.2)
7.9.	Comments on the external validation of the model	Highly predictive
**8**	**Providing a mechanistic interpretation - OECD Principle 5: "A MECHANISTIC INTERPRETATION, IF POSSIBLE"**	**PRINCIPLE 5: "A MECHANISTIC INTERPRETATION, IF POSSIBLE". According to PRINCIPLE 5, a (Q)SAR should be associated with a mechanistic interpretation, if possible.**
8.1.	Mechanistic basis of the model	-NA- since the final models are stacked models that use the predicted values of individual MLR q-RASPR models as descriptors and not the structural, physicochemical, and periodic table descriptors.
8.2.	A priori or a posteriori mechanistic interpretation	-NA-
8.3.	Other information about the mechanistic interpretation	-NA-
**9**	**Miscellaneous information**	
9.1.	Comments	None

9.2.	Bibliography	1. Srisongkram T. Chem Res Toxicol 2023, 36, 1961-1972.<br>2. Alexander DLJ, Tropsha A, Winkler DA. J Chem Inf Model 2015, 55, 1316-1322.
9.3	Supporting information	The training and test datasets used to develop stacked PLS and MLP q-RASPR models have been attached in the Supplementary Information.
